# Latent and active aurone synthase from petals of *C. grandiflora*: a polyphenol oxidase with unique characteristics

**DOI:** 10.1007/s00425-015-2261-0

**Published:** 2015-02-20

**Authors:** Christian Molitor, Stephan Gerhard Mauracher, Sanela Pargan, Rupert L. Mayer, Heidi Halbwirth, Annette Rompel

**Affiliations:** 1 Institut für Biophysikalische Chemie, Fakultät für Chemie, Universität Wien, Althanstraße 14, 1090 Vienna, Austria; 2 Department of Analytical Chemistry, University of Vienna, Währinger Straße 38, 1090 Vienna, Austria; 3 Institute of Chemical Engineering, University of Technology Vienna, Getreidemarkt 9, 1060 Vienna, Austria

**Keywords:** Allosteric activation, Aurone synthase, 4-Deoxyaurone, Polyphenol oxidase, Proteolytic activation, Type-3 copper enzyme

## Abstract

**Aurone synthase belongs to the novel group 2 polyphenol oxidases and the presented kinetic characterization suggests a differing aurone biosynthesis in Asteraceae species compared to snapdragon.**

Aurone synthases (AUS) are polyphenol oxidases (PPO) physiologically involved in the formation of yellow aurone pigments in petals of various Asteraceae species. They catalyze the oxidative conversion of chalcones into aurones. Latent (58.9 kDa) and active (41.6 kDa) aurone synthase from petals of *C. grandiflora* was purified by a quantitative removal of pigments using aqueous two-phase separation and several subsequent chromatographic steps. The purified enzymes were identified as *cg*AUS1 (A0A075DN54) and sequence analysis revealed that *cg*AUS1 is a member of a new group of plant PPOs. Mass determination experiments of intact *cg*AUS1 gave evidence that the C-terminal domain, usually shielding the active site of latent polyphenol oxidases, is linked to the main core by a disulfide bond. This is a novel and unique structural feature of plant PPOs. Proteolytic activation in vivo leads to active aurone synthase possessing a residual peptide of the C-terminal domain. Kinetic characterization of purified *cg*AUS1 strongly suggests a specific involvement in 4-deoxyaurone biosynthesis in *Coreopsis grandiflora* (Asteraceae) that differs in various aspects compared to the 4-hydroxyaurone formation in *Antirrhinum majus* (Plantaginaceae): *cg*AUS1 is predicted to be localized in the thylakoid lumen, it possesses exclusively diphenolase activity and the results suggest that aurone formation occurs at the level of chalcone aglycones. The latent enzyme exhibits allosteric activation which changes at a specific product concentration to a constant reaction rate. The presented novel structural and functional properties of aurone synthase provide further insights in the diversity and role of plant PPOs.

**Electronic supplementary material:**

The online version of this article (doi:10.1007/s00425-015-2261-0) contains supplementary material, which is available to authorized users.

## Introduction

**Fig. 1:**
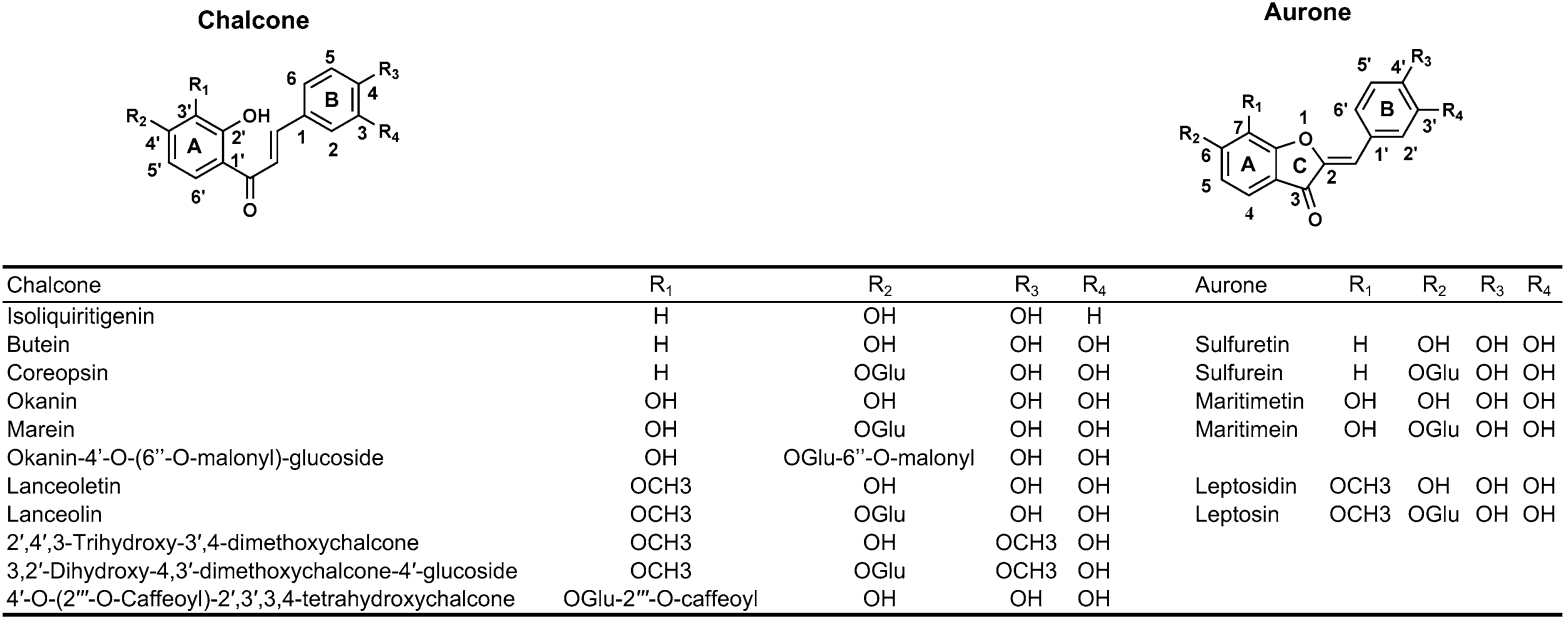
6′-deoxychalcones and 4-deoxyaurones identified in petals of *C. maritima*, *C. gigantea*, *C. bigelovii*, *C. grandiflora*, *C. mutica*, *C. tinctoria*, *C. saxicola* and *C. lanceolata* (Geissman and Heaton 1943, 1944; Geissman and Mojé 1951; Shimokoriyama and Hattori 1953; Geissman et al. 1956; Harborne and Geissman 1956; Crawford 1970; Nicholls and Bohm 1979; Crawford and Smith 1980, 1983; Tanimoto et al. 2009; Shang et al. 2013; Okada et al. 2014; Wang et al. 2014)

Chalcones and aurones are yellow plant pigments related to flavonoids. They are called anthochlor pigments and are found in yellow flowers of Asteraceae species, snapdragon (*Antirrhinum majus L.*) and carnations (*Dianthus caryophyllus*) (Harborne 1967). Two types of anthochlors exist distinguished by the presence or absence of a hydroxyl group in ring A at position 6′ of chalcones and position 4 of aurones (note differing numbering of chalcones and aurones, Fig. 1). In Asteraceae, anthochlor pigments of the deoxy type are typically found. Several 6′-deoxychalcones and 4-deoxyaurones (Fig. 1) were identified in extracts of petals of the genus of Coreopsis (*C. maritima*, *C. gigantea*, *C. bigelovii*, *C. grandiflora*, *C. mutica*, *C. tinctoria*, *C. saxicola* and *C. lanceolata*) (Geissman and Heaton 1943, 1944; Geissman and Mojé 1951; Shimokoriyama and Hattori 1953; Geissman et al. 1956; Harborne and Geissman 1956; Crawford 1970; Nicholls and Bohm 1979; Crawford and Smith 1980, 1983; Tanimoto et al. 2009; Shang et al. 2013; Okada et al. 2014; Wang et al. 2014). In contrast to the extensively studied flavonoid formation, knowledge about aurone biosynthesis is still very limited.

Aurone formation was studied primarily in *Antirrhinum majus* (snapdragon) as a model plant for the formation of hydroxyanthochlors. A homologue of polyphenol oxidases (PPOs), aureusidin synthase from *A. majus* (*Am*AS1), was identified as the key enzyme in aurone biosynthesis in snapdragon (Nakayama et al. 2000) as one of the rare examples of a PPO involved in an anabolic pathway (Strack and Schliemann 2001).

PPOs are type-3 copper enzymes which are widespread among plants, fungi and bacteria displaying a broad range of putative physiological roles, primarily in the protection of organisms against biotic and abiotic stress. A specific involvement in biosynthetic processes, beside aurone biosynthesis, was shown only in a few cases (Mayer 2006; Wahler et al. 2009; Araji et al. 2014). Plant PPOs are expressed in their latent state, in which the active site of the enzyme is shielded by the C-terminal domain, which is ultimately removed by proteolytic cleavage resulting in a fully active enzyme (King and Flurkey 1987; Espin et al. 1999; Gandia-Herrero et al. 2005; Marusek et al. 2006; Flurkey and Inlow 2008). It was shown that the latent pro-enzymes undergo allosteric activation with several substrates (Valero and Garcia-Carmona 1992, 1998; Nillius et al. 2008). In vitro, the pro-enzymes can be activated by proteases, an acidic pH, fatty acids or detergents (e.g. SDS) (Yoruk and Marshall 2003).

PPOs comprise monophenolases [tyrosinases (EC 1.14.18.1)], catalyzing the *o*-hydroxylation of monophenols and oxidation of *o*-diphenols to *o*-quinones, and diphenolases [catechol oxidases (EC 1.10.3.1)], which lack monophenolase activity and catalyze the oxidation reaction exclusively. Although PPOs have been studied over many decades and crystal structures of tyrosinases and catechol oxidases are available, no prediction of their reactivity based on sequence information has been possible so far. During catalysis, PPOs can undergo suicide inactivation, which is proposed to be caused by a substrate docking mode that leads to the reductive loss of copper (Land et al. 2008; Muñoz-Muñoz et al. 2008, 2011, 2012a, b; Ramsden et al. 2009; Ramsden and Riley 2010a).

Most plant PPOs contain an N-terminal chloroplast transit peptide (cTP) and a thylakoid transfer domain and are hence predicted to be transported to the thylakoid lumen in the chloroplast. Only a few PPOs lack the cTP, but instead contain a N-terminal secretion signal peptide (Tran et al. 2012). Apart from PPO from *Populus trichocarpa,* *Am*AS1 is the only PPO for which a vacuolar localisation was demonstrated (Ono et al. 2006b; Tran and Constabel 2011). In accordance with its presence in vacuoles, *Am*AS1 was found to be a glycoprotein (Nakayama et al. 2000). Notably, a chalcone 2′-*O*-glycosyltransferase is required for the accumulation of aurones in transgenic plants (Nakayama 2002; Shakya et al. 2012) because transport into vacuoles occurs only on extensively modified structures and not on aglycones (Matern et al. 1986).

Recently, it was suggested that the formation of 4-deoxyaurones differs from that of 4-hydroxyaurones in various aspects. Most notably, a PPO homologue lacking the monophenolase activity appears to be responsible for aurone formation (Miosic et al. 2013; Kaintz et al. 2014), while the introduction of a second vicinal hydroxyl group in the B-ring of chalcones is catalyzed in an independent step by a chalcone 3-hydroxylase (Schlangen et al. 2010). Recently, two cDNA clones (*cgAUS1* (KC972611) and *cgAUS2* (KC878307)) of putative aurone synthases from petals of *Coreopsis grandiflora* (*cg*AUS) were isolated (Kaintz et al. 2014). *cg*AUS1 and *cg*AUS2 showed divergent gene expression profiles. *cg*AUS1 was particularly expressed in petals, as expected for a gene involved in the biosynthesis of flower pigments. In contrast, *cg*AUS2 displayed higher expression rates in green leaves rather than in flowers. Both gene products are predicted to be subcellularly localized in the chloroplasts and possess highly conserved motifs of plant PPOs (Kaintz et al. 2014).

To verify the putative involvement of *cg*AUS1 in aurone biosynthesis in *C. grandiflora,* we purified high amounts of active as well as latent *cg*AUS from petals for characterization and subsequent crystallization experiments. The purified PPO, identified as *cg*AUS1 (A0A075DN54), is a member of the novel group 2 PPOs and possesses hitherto unknown structural and functional properties. The substrate specificity of *cg*AUS1 is compared with a common catechol oxidase from *Vitis vinifera* (*vv*CO). Based on our findings, we propose an aurone biosynthetic pathway in Asteraceae species alternative to the one reported for *A. majus* (Davies et al. 2006; Ono et al. 2006a).

## Materials and methods

### Plant material

Seedlings of *C. grandiflora* cv. Early sunrise were purchased from Volmary (Münster, Germany), cultivated in April 2011 in the experimental field Augarten of the University of Vienna (Austria). Petals of all developmental stages were harvested from June to September, all green tissues were discarded and the yellow plant material was shock-frozen in liquid nitrogen and stored at −80 °C.

### Reagents

Butein and marein were purchased from Extrasynthesis (Genay, France). All other used chemicals were purchased from Sigma-Aldrich at the highest purity available.

### Purification, yield and SDS-PAGE of catechol oxidase from *Vitis vinifera* (*vv*CO)

Catechol oxidase from *V. vinifera* was used as a reference enzyme to evaluate substrate specificity of aurone synthase. The description of the isolation, purification, yield and SDS-PAGE analysis of this enzyme is presented in Online Resource Fig. S1.

### Protein extraction and removal of pigments

The pigment removal by an aqueous two-phase separation (ATPS) procedure described below is based on the methods described by Sojo et al. (1998), which were, however, significantly modified and optimized in several aspects. Similar procedures were also reported for the isolation of tyrosinase from *Juglans regia* (Zekiri et al. 2014) and *Agaricus bisporus* (Mauracher et al. 2014). Protein extraction was carried out on 6 kg petal tissue in total which was processed in three batches as outlined below.

2 kg of frozen petals were homogenized with a hand-held blender. 4 l buffer containing 125 mm sodium citrate, pH 5.4, 4 % (v/v) Triton X-114, 0.5 % (w/v) sodium ascorbate, 50 mm l-proline, 1 mm phenylmethylsulfonyl fluoride (PMSF) and 2 mm benzamidine hydrochloride were added and the pulp was centrifuged at 16,000*g* for 20 min at 4 °C. The supernatant was filtered through cheesecloth, 15 g/l ammonium sulfate was added and the solution was warmed up to 8–12 °C. This caused the solution to become turbid due to micelle growth and subsequent liquid-liquid phase separation. After centrifugation at 16,000*g* for 10 min at 12 °C, the brownish detergent-rich bottom phase was discarded and the brighter detergent-poor top phase was supplemented with Triton X-114 to a final concentration of approx. 4 % (v/v). The turbid solution was stirred for 15 min at 8–12 °C, and was then centrifuged at 16,000*g* for 10 min at 12 °C. The bottom phase was again discarded and ammonium sulfate was added to a final concentration of 30 % saturation to the orange top phase. After stirring to dissolve the salt, the suspension was allowed to chill at 4 °C for 45 min. All the following steps were performed at 4 °C. After centrifugation at 16,000*g* for 45 min and filtration, PEG-4000 was added to a final concentration of 4.5 % (w/v). The turbid solution was centrifuged at 16,000*g* for 10 min and the yellow–orange colored detergent-rich top phase was discarded, while the lower detergent-poor phase was subjected to another round of ATPS. This procedure was repeated 3–5 times with descending additions of PEG-4000 (4, 3.5, 3 % (w/v), respectively), until the red to yellow colored pigments were removed quantitatively. To the resultant clear and slightly beige colored enzyme solution ammonium sulfate was added to a final concentration of approx. 85 % saturation and stored at 4 °C over night. The ammonium sulfate solution beneath the floating pellet was discarded with the pellet transferred to a bottle-top filter (GE Healthcare, PES, 45 µm) to reduce the ammonium sulfate solution content to a minimum via gravity-driven filtration.

### Enzymatic assay

AUS assays of initial purifications were performed by monitoring the increase in absorbance at 415 nm due to the conversion of butein into sulfuretin. The reaction mixture contained 40 µm butein in 125 mm sodium citrate at pH 5.5.

Enzymatic assays for subsequent purifications were performed by monitoring the oxidation of 50 µm fisetin in 125 mm sodium citrate at pH 5.5 at 280 nm (Jimenez et al. 1998). When allosteric activation during the enzymatic reaction was visible, the fractions contained portions of latent protein. Further assays for the latent forms were performed either by adding 2.5 mm SDS to the assay mixture or using 125 mm sodium citrate at pH 3.5 to activate the protein to obtain a linear absorbance–time relation.

### Kinetic analysis

Experiments for determination of kinetic parameters of aurone synthase were performed using sample 1 (*cg*AUS1) while varying the substrate concentration. For evaluation of the substrate specificity of aurone synthase, kinetic parameters of a purified catechol oxidase from *Vitis vinifera* (*vv*CO) were determined as well. Mean values and standard deviations were calculated based on four repetitions. Data analysis was carried out by nonlinear regression (Marquardt 1963). In cases where an accurate estimation of *K*_m_ by nonlinear fitting was not possible due to the low solubility of substrates, the ratio *k*_cat_/*K*_m_ was determined by linear fitting to pseudo first-order kinetics ([S] ≪ *K*_m_). Separations of chalcones and aurones applying HPLC were performed as previously described by Miosic et al. (2013) using the protocol of Chandra et al. (2001).

Purified heterologously expressed *cg*AUS1 (Kaintz et al. 2014) exhibited properties identical to the latent *cg*AUS1 purified from the natural source. Due to traces of active *cg*AUS1 in the sample purified from the natural source, recombinantly expressed *cg*AUS1 was used to describe the characteristics of the latent enzyme.

### Protein purification by Fast Protein Liquid Chromatography (FPLC)

All chromatographic purification steps were carried out using an *Äkta Purifier* (*GE Healthcare*) placed in a refrigerator to maintain 4 °C.

An appropriate portion of the drained protein pellet obtained was dissolved in 300 ml 20 mm sodium acetate buffer (pH 5.0), centrifuged at 28,000*g* for 45 min at 4 °C and further diluted with buffer until the conductivity of the enzyme solution was decreased below 11 mS/cm. The solution was applied to a SP-Sepharose FF column (HiScale 26/40, 70 ml bed volume) via a sample pump at a flow rate of 13 ml/min. Proteins were eluted by an increasing sodium chloride gradient over 15 column volumes (CV) up to 0.8 m sodium chloride at a flow rate of 5 ml/min. Although the majority of the resulting fractions contained AUS activity, slight maxima in activity could be determined. This was taken into account for pooling of the active fractions.

The pooled fractions were diluted by a factor of three with 20 mm sodium acetate (pH 5.0) and applied to a SP-Sepharose HP column (XK 16, 10 ml bed volume) at a flow rate of 5 ml/min. Elution was achieved by an increasing sodium chloride gradient over 10 CV, up to 0.8 m at a flow rate of 3 ml/min. Active fractions were pooled, concentrated by ultracentrifugation (30 kDa molecular weight cut off, MWCO), diluted in 45 ml 30 mm Tris/HCl buffer (pH 8.5) and loaded on a Mono Q HR 5/50 GL column. Proteins were eluted by an increasing sodium chloride gradient up to 250 mm sodium chloride over 200 CV. Several related fractions, showing either ordinary activity or allosteric behavior towards fisetin as substrate, were pooled, concentrated by ultracentrifugation (30 kDa MWCO), diluted in 45 ml 20 mm sodium acetate (pH 5.0) and loaded onto a Mono S HR 5/50 GL column. Elution was performed by applying an increasing sodium chloride gradient up to 500 mm over 200 CV. Selected active and latent fractions were again subjected to Mono S cation exchange chromatography at pH 5.0, whereas, due to overlapping protein peaks, a change to 20 mm sodium citrate (pH 5.6) was necessary as a polishing step for one latent form.

A buffer exchange to 10 mm sodium acetate (pH 5.0) of the purified fractions was performed by ultracentrifugation (10 kDa MWCO) and the concentrated samples (6–10 mg/ml) were stored at 4 °C.

### Molecular mass determination

SDS-PAGE was performed according to the method of Laemmli (1970), using Precision Plus Protein Standard Dual Color (Bio-Rad) as the molecular weight marker. The samples were applied to 10 % polyacrylamide gels under reducing and non-reducing conditions by adding or excluding dl-dithiothreitol (DTT). Gels were stained with Coomassie Brilliant Blue R-250.

Electrospray Ionization Mass Spectrometry (ESI–MS) was performed on a nanoESI-QTOF mass spectrometer (maXis 4G UHR-TOF, Bruker) with a mass resolving power of about 40,000 in the used m/z—range and a mass accuracy of better than 5 ppm (confirmed by standard proteins) using a protein solution of 20 µl with a concentration of approximately 1 µg/µl. Buffer exchange to 10 mm ammonium acetate (pH 5.0) of non-reduced samples was performed by ultracentrifugation. After buffer exchange to 20 mm ammonium acetate (pH 7.5) by ultracentrifugation of samples for measurements under reducing conditions, DTT was added to a final concentration of 50 mm. Subsequently, the samples were incubated for 45 min at 50 °C. Just prior to the measurements, acetonitrile to a final concentration of 25 % (v/v) and formic acid to a final concentration of 0.05 % (v/v) were added.

### Protein identification

Tryptic digestions of enzyme solutions containing approximately 10 µg protein were performed according to the manufacturers’ description. The peptide samples were dried by vacuum centrifugation and stored at -20 °C for LC–MS/MS analysis. The samples were solubilized in 5 µl 30 % (v/v) formic acid and diluted with 40 µl 2 % (v/v) acetonitrile, 0.1 % (v/v) formic acid.

Analysis of the samples was carried out by nanoUHPLC–ESI–MS/MS using a high-resolution orbitrap mass spectrometer (Dionex Ultimate 3000 RSLCnano, Q Exactive orbitrap, Thermo Scientific). The data analysis was performed with Proteome Discoverer 1.4 by searching against the sequences of *cg*AUS1 (A0A075DN54) and *cg*AUS2 (A0A075BWS7). The search engine applied was Sequest. The peptide mass tolerance was 5 ppm and the fragment mass tolerance 0.5 Da. Carbamidomethylation of cysteines was set as a static modification, whereas phosphorylation of serine, threonine and tyrosine as well as sulfation of tyrosine were set as variable modifications. For high confidence in the MS data, the false discovery rate (FDR) of the peptide spectrum matches (PSM) was set to <0.01 (Proteome Discoverer). The search results were filtered with cross-correlation (XCorr) scores, set as 2.0, 2.5, 3.0 and 3.5 for peptides carrying 1, 2 and 3 or 4 charges, respectively.

## Results

### Protein extraction and removal of pigments

Petals from all developmental stages of flowers show AUS activity (Kaintz et al. 2014). To obtain a high amount of aurone synthase, 6 kg petal tissue (3 batches of 2 kg) of all developmental stages was used as starting material. Pigments were quantitatively removed from the crude extract by a combination of repeated Triton X-114-induced ATPS, ammonium sulfate precipitation and iterated PEG-4000-induced ATPS. The addition of ammonium sulfate to the crude extract containing Triton X-114 at the given pH lowered the cloud point to temperatures (8–12 °C) that are less harsh for proteins in comparison to the temperature induced ATPS at 37 °C (Sojo et al. 1998). The consecutive addition of ammonium sulfate to 30 % saturation led to further removal of pigments by precipitation. Pigments still present were removed by PEG-4000-induced ATPS with descending additions of PEG-4000 to avoid protein precipitation. A clear, beige to colorless enzyme solution was finally obtained.

### Measurement of enzyme activity during protein purification

During the optimization of the purification procedure, the chalcone butein (Fig. 1) and the flavonol fisetin (Jimenez et al. 1998), both possessing two vicinal hydroxyl groups in ring B, were used in spectrophotometrical assays of the obtained fractions. No differences in substrate specificity between different fractions were observed. However, some fractions showed a sigmoid time course when fisetin was used as a substrate indicating an allosteric activation mechanism (Valero and Garcia-Carmona 1992). Fractions showing this behavior were, therefore, considered to contain portions of latent *cg*AUS. Modification of the enzymatic assays either by addition of 2.5 mm SDS or by applying a pH of 3.5 lead to a steady-state reaction rate.

### Protein purification by Fast Protein Liquid Chromatography (FPLC)

**Fig. 2:**
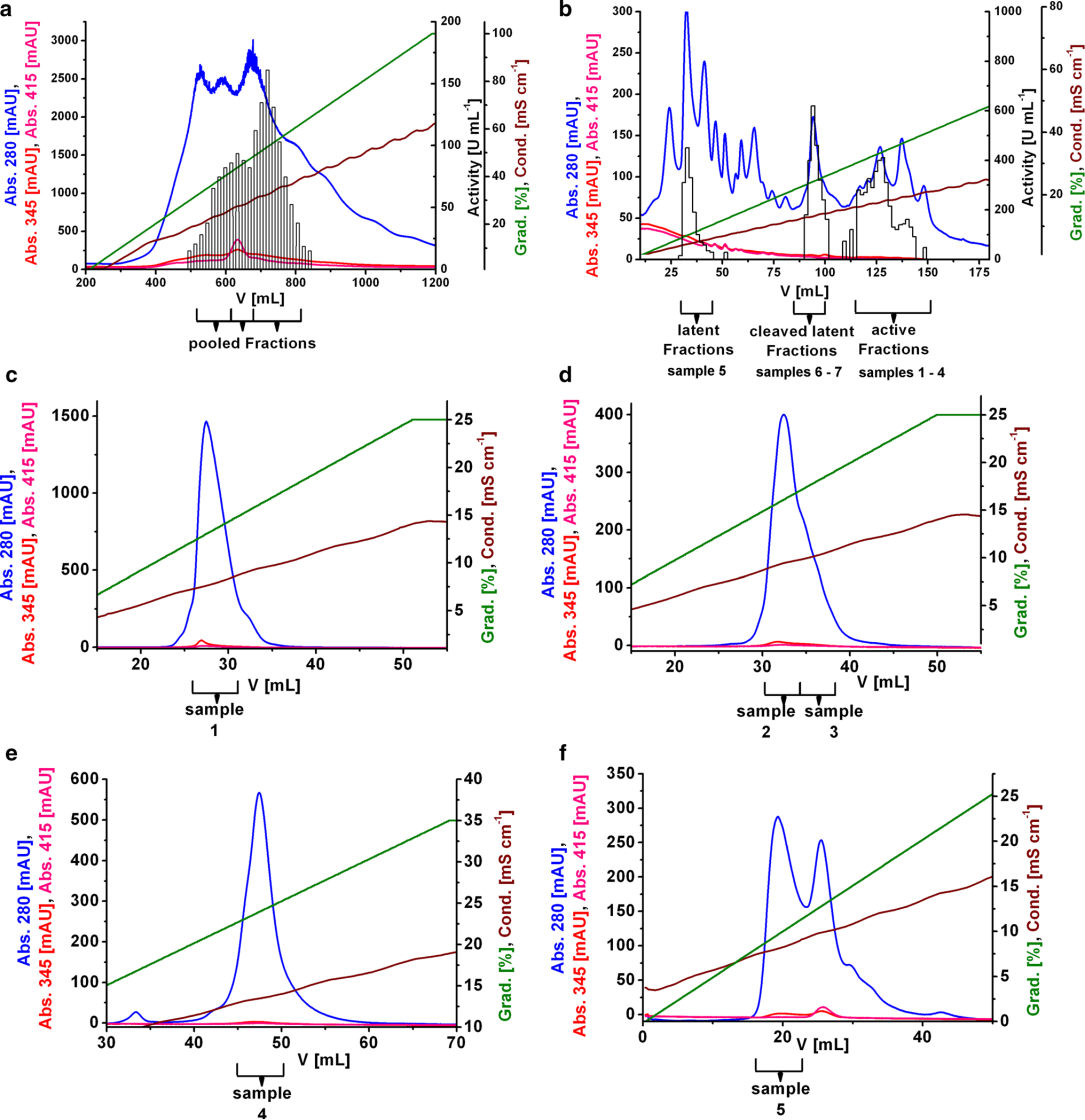
Typical chromatograms of the purification procedure of latent and active *cg*AUS. Enzymatic assays for active *cg*AUS were performed by monitoring the oxidation of 1 ml 50 µm fisetin in 125 mm sodium citrate pH 5.5 at 280 nm (Jimenez et al. 1998). 2.5 mm SDS were added to the reaction mixture for latent and proteolytically cleaved latent *cg*AUS assays. One unit of enzyme was defined as the amount that catalyzed the formation of 1 nmol of oxidized fisetin per minute. **a** CEX chromatography using SP-Sepharose FF, pH 5.0. **b** AEX chromatography using Mono Q, pH 8.5. Fractions containing latent, proteolytically cleaved latent and active *cg*AUS are indicated below the chromatogram. **c** Polishing CEX chromatography of active sample 1 using Mono S, pH 5.0. **d** Polishing CEX chromatography of active samples 2 and 3 using Mono S, pH 5.0. **e** Polishing CEX chromatography of active sample 4 using Mono S, pH 5.0. **f** Polishing CEX chromatography of latent sample 5 using Mono S, pH 5.6

The chromatogram obtained by cation exchange (CEX) chromatography on SP-Sepharose FF showed broad activity peaks (Fig. 2a). The occurrence of several active forms made it necessary to perform 5 purification steps (6 for the latent form). Due to limited column capacities and sample shelf life, several interlaced purification lines were performed in parallel. Related forms of identical purification stages were combined guided by activity and conductivity of the eluted fractions. In total, seven different samples containing significant amounts of *cg*AUS, in total approximately 5.9 mg, (Table 1) were purified and characterized. Samples 1–4 contained active *cg*AUS and samples 5–7 contained latent *cg*AUS. Latent (sample 5) and cleaved latent forms (samples 6–7) were separated on the stage of Mono Q from the active forms (samples 1–4) (Fig. 2b). Example chromatograms of the purification on SP-Sepharose FF, Mono Q and the polishing steps on Mono S of the samples 1–5 are shown in Fig. 2.

**Table 1:** Yields, molecular masses (SDS-PAGE) and the kinetic classification of purified *cg*AUS samples. 6 kg frozen petal material was used as starting material

Sample	Amount (mg)	M (kDa) (reducing SDS-PAGE)	M (kDa) (non-reducing SDS-PAGE)	Latent/active
1	2.45	37; 38	36; 37; 38	Active
2	0.56	37; 38	36; 37; 38	Active
3	0.37	37; 38	36; 37; 38	Active
4	1.25	37; 38	36; 37; 38	Active
5	0.87	59	65, 61, 54	Latent
6	0.25	38; 19	65, 54	Latent
7	0.12	37; 19	65, 54	Latent

Molecular masses were determined by reducing and non-reducing SDS-PAGE. Satellite bands exhibiting a shift to lower masses are found due to incomplete denaturation and/or incomplete reduction of the applied samples

### Gel electrophoresis and mass determination by ESI–QTOF–MS

**Fig. 3:**
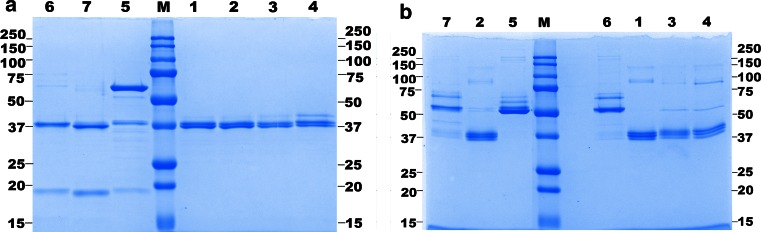
SDS-PAGE analysis of purified *cg*AUS. About 4 µg total protein was loaded on each lane. Lane-numbers in **a** and **b** correspond to the described *cg*AUS samples within this article. Lane-number 1–4: active *cg*AUS; lane-number 5: latent *cg*AUS; lane-numbers 6 and 7: proteolytically cleaved latent *cg*AUS; M, molecular weight marker. **a** SDS-PAGE (according to Laemmli 1970) of purified *cg*AUS. **b** SDS-PAGE of purified *cg*AUS under semi-denaturating conditions: Laemmli sample buffer without DTT, samples were not boiled

**Fig. 4:**
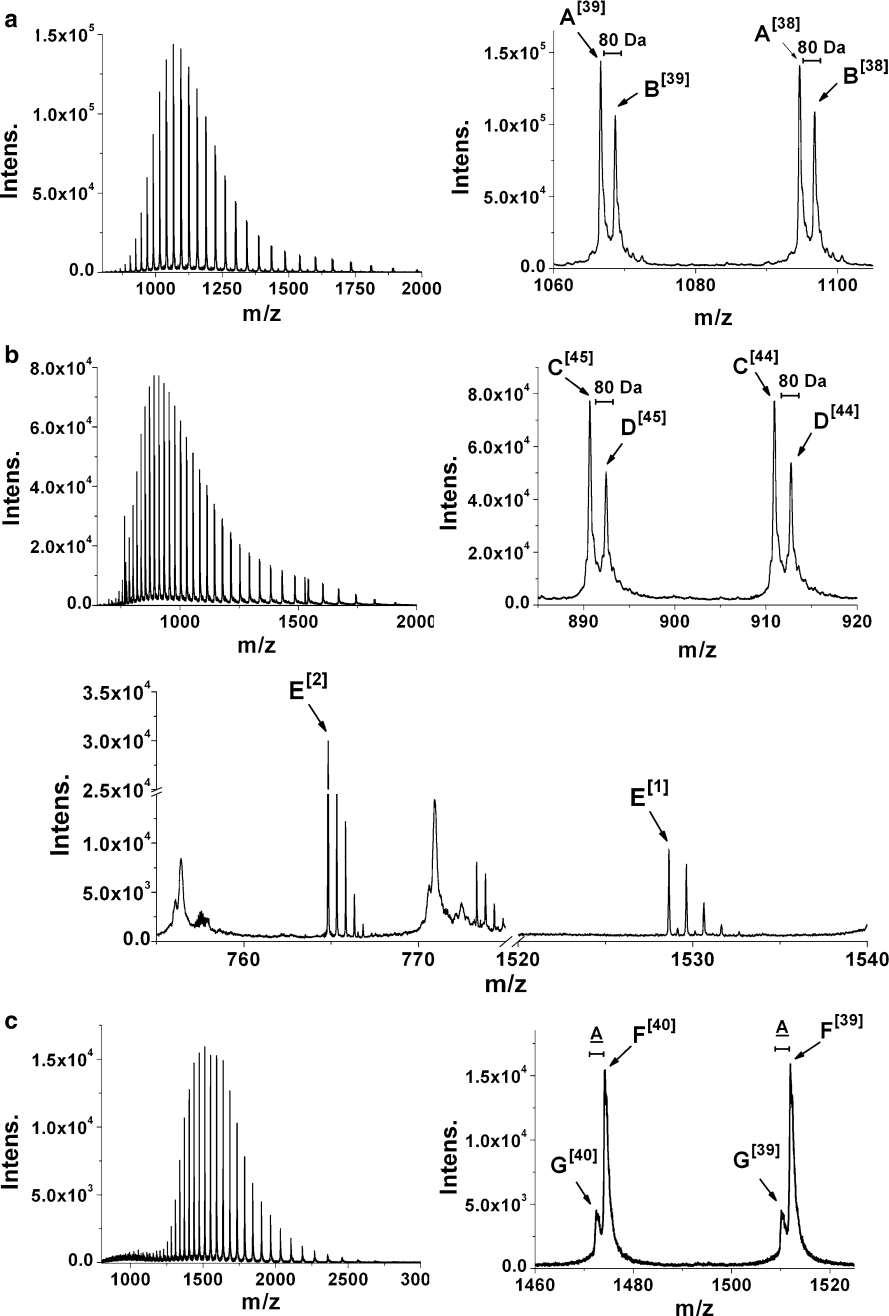
Positive mode ESI-QTOF mass spectra of purified active and latent *cg*AUS1 samples. Entire and magnified mass spectra of acidified sample 1 containing active *cg*AUS1. **a** Under non-reducing conditions (untreated). **b** Under reducing conditions (preincubated with 50 mm DTT). **c** Mass spectra of sample 5 containing acidified latent *cg*AUS1 under non-reducing conditions

**Fig. 5:**
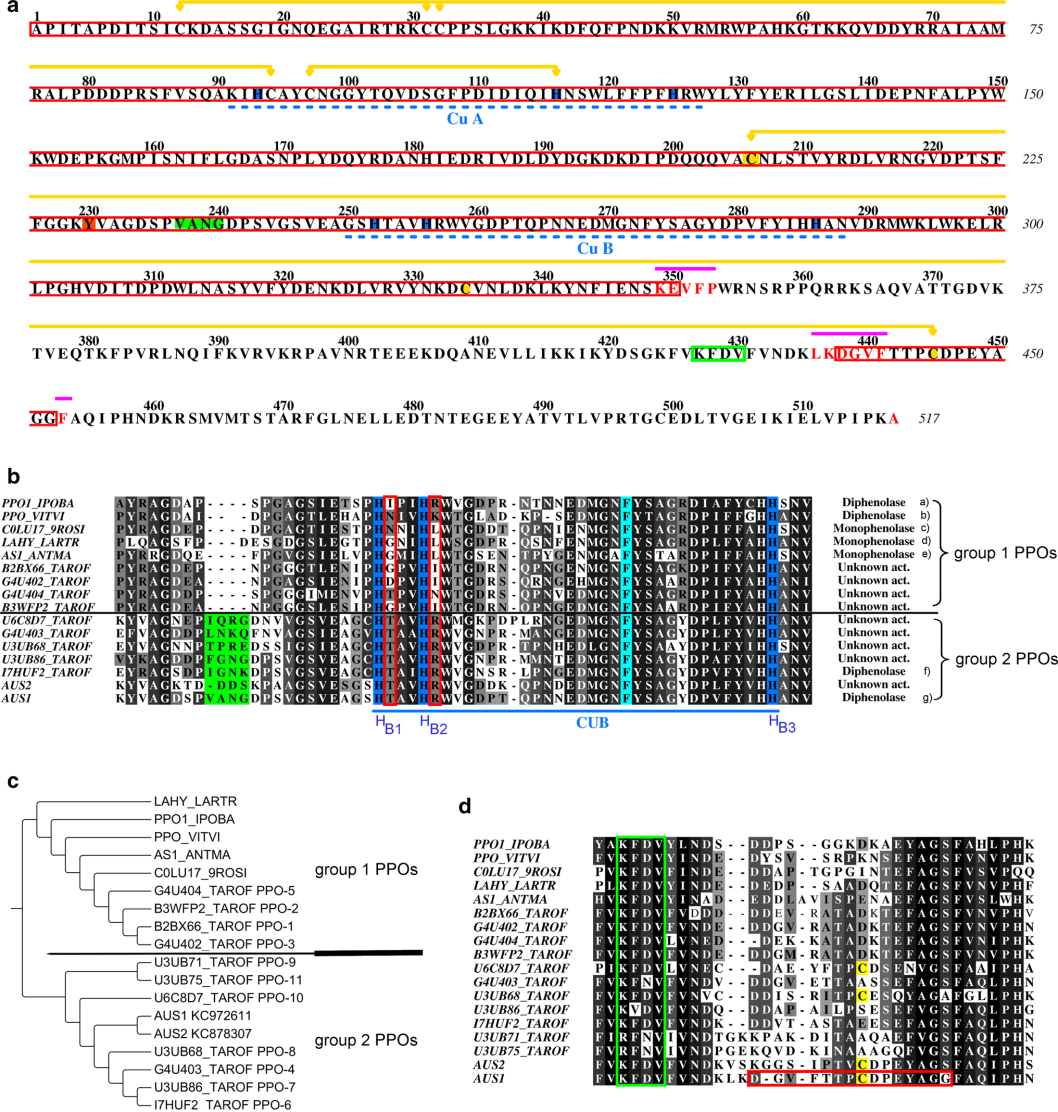
Sequence analysis and phylogenetic tree of *cg*AUS1 in comparison with other PPOs. **a** Primary structure of *cg*AUS1 excluding the transit peptide. *Red rectangles* are used in the alignment to highlight the deduced primary structure of the sample 1 whereas terminal amino acids of species of minor abundance (compare ESI-Q-TOF measurements, Fig. S2; Table S1) are written in a *red color* font. Copper-binding histidines are highlighted *blue*, copper-binding sites are *underlined* with *blue* *dashed lines*. Proteolytic cleavage sites are marked by *violet lines*. Disulfide linkages and the thioether-bridge are marked by yellow connectors. The phosphorylated/sulfated tyrosine residue (identified by HPLC–ESI–MS/MS experiments) is highlighted in *orange*. The insertion in the loop region near the active site of catechol oxidases (PPO_VITVI pdb-entry: 2P3X; PPO1_IPOBA pdb-entry: 1BT3) is colored in *green* and the highly conserved KFDV-motif is marked by a *green rectangle*. **b** Sequence alignments of CuB binding sites of several plant PPOs (monophenolase/diphenolase activity: (a) Klabunde et al. 1998, (b) Virador et al. 2010, (c) Escobar et al. 2008; Zekiri et al. 2014, (d) Cho et al. 2003, (e) Nakayama et al. 2000, (f) Dirks-Hofmeister et al. 2012, (g) this work). The copper-binding histidines are highlighted *blue*, the conserved phenylalanine above the active site is highlighted in *pale blue*, the insertion of group 2 PPOs in the loop region near the active site is highlighted in *green*. For clarity the sequences of dandelion PPO-9 and PPO-11 were omitted, because the insertion is even larger. The positions H_B1_+1 and H_B2_+1 are marked by *red rectangles*. **c** Phylogenetic tree of several plant PPOs. The corresponding alignment of these PPOs is presented in Online Resource Fig. S3. **d** Sequence alignments near the C-terminal residual peptide found to be connected by a disulfide linkage to the main core of mature *cg*AUS1. The KFDV-motif is highlighted with *green rectangles* and the cysteines are shaded in *yellow*. The primary structure of *cg*AUS1 and sequence alignments were prepared using ALINE (Bond and Schuttelkopf 2009). The phylogenetic tree was prepared using TreeGraph 2 (Stover and Muller 2010)

The gels showed two bands for active *cg*AUS samples after reducing and three bands after non-reducing SDS-PAGE at a molecular weight of approx. 37 kDa (Fig. 3a, b, lane-numbers 1–4, Table 1). The occurrence of several bands is caused by incomplete denaturation and incomplete reduction of the protein, evidenced by ESI–QTOF–MS molecular mass determination (Fig. 4; Table 2, Online Resources Fig. S2; Table S1). Incomplete denaturation is also visible for the latent *cg*AUS samples under non-reducing conditions. The latent *cg*AUS (sample 5) showed a mass of 54 kDa and 59 kDa under non-reducing and reducing conditions (Fig. 3a, b, lane-number 5). Interestingly, the latent samples 6 and 7 showed masses of the latent *cg*AUS under non-reducing conditions, but molecular masses of the active *cg*AUS under reducing conditions (Fig. 3a, b, lane-numbers 6 and 7, Table 1). An additional band at about 19 kDa occurred under reducing conditions, which indicates that the C-terminal domain is bound to the main core by a disulfide bridge. This hypothesis was supported by mass determination of active *cg*AUS samples 1–4 (compare Table 1) by ESI–QTOF–MS under reducing and non-reducing conditions. The obtained mass spectra are shown in Fig. 4 and in Online Resource Fig. S2. The mass spectrum of *cg*AUS sample 1 under non-reducing conditions showed two major species exhibiting masses of 41,559.0 and 41,639.0 Da, with an intensity ratio of about 1.4:1 and a mass difference of 80.0 Da (Fig. 4a, species A and B). Several peaks with significantly lower intensities, each exhibiting mass differences of approximately 16 Da, were also present for the two main peaks, indicating that the protein might contain several oxidized residues. The mass difference of 80 Da may be caused by either a phosphorylation or a sulfation. Under reducing conditions species A was shifted by −1,523.4 Da and species B by −1,523.3 Da (Fig. 4b, species C and D), with a peptide of the corresponding mass (E^1^, E^2^) appearing. The monoisotopic mass matched the calculated value of the sequence D^438^GVFTTPCDPEYAGG^452^ of *cg*AUS1. This sequence is localized downstream of the main core in the C-terminal region of the pro-enzyme (Fig. 5a). The existence of a disulfide bridge connecting the main core of *cg*AUS1 with the shielding C-terminus is thus supported by the ESI–QTOF–MS experiments.

**Table 2:** Masses determined from deconvoluted mass spectra of active and latent *cg*AUS1 under reducing and non-reducing conditions

Sample	Species	M (exp.) (Da)	Deduced sequence	M (calc.) [Da]
1	A	41,559.0 ± 0.3	API−IENSKE//DGVFTTPCDPEYAGG	41,559.35
	B	41,639.0 ± 0.4	API−IENSKE + X//DGVFTTPCDPEYAGG	41,639.35
1 reduced	C	40,035.6 ± 0.3	API−IENSKE	40,036.79
	D	40,115.7 ± 0.3	API−IENSKE + X	40,116.79
	E	764.8207 [M + 2H]^2+^ (mono) 1,528.6318 [M + H]^+^ (mono)	DGVFTTPCDPEYAGG	764.8167 [M + 2H]^2+^ (mono) 1,528.6261 [M + H]^+^ (mono)
5	F	58,928.2 ± 0.6	API−PIPKA	58,927.37
	G	58,858.0 ± 1.7	API−PIPK	58,856.29

Theoretical masses were calculated by including the two disulfide bridges, one thioether-bridge [existence assumed by similarity to plant catechol oxidases PPO_VITVI (P43311) and PPO1_IPOBA (Q9ZP19)], the previously unknown disulfide bond linking the C-terminus to the main core as well as the C-terminally hydrolyzed peptide backbone

*X* is assumed to have a molecular weight of 80.0 Da

Theoretical masses for the *cg*AUS1 sequence (A0A075DN54) were calculated by taking into account 3 disulfide linkages (−6H), the thioether crosslink (−2H) as well as the C-terminally hydrolyzed peptide backbone (+H_2_O). Based on these calculations the primary structure of the active *cg*AUS1 (sample 1) was deduced (Table 2; Fig. 5a, the primary structure is highlighted by red rectangles).

The mass spectra of active *cg*AUS samples 2–4 (Online Resource Fig. S2) showed several peaks. The peaks that differ in 80 Da from each other show intensity ratios comparable to the observed ratios of sample 1. Calculations of theoretical masses of *cg*AUS1 showed that the mass differences between the different species are caused by a combination of a phosphorylation/sulfation and unspecific proteolytic cleavage occurring at the C-terminus of the main core and at the remaining C-terminal peptide (Online Recource Table S1). The terminal amino acids of these species are indicated by a red color font in Fig. 5a.

The mass spectrum of latent *cg*AUS (sample 5) under non-reducing conditions displayed two peaks with an intensity ratio of 1:3.6 (Fig. 4c). Charge deconvolution yielded masses of 58,928.2 Da, matching the mass of latent *cg*AUS1, and 58,858.0 Da, respectively. The resulting mass difference can be explained as a cleaved alanine, most likely caused by proteolytic cleavage of A^517^ (Table 2; Fig. 5a).

### Protein identification

Identification of the purified enzyme as *cg*AUS1 (A0A075DN54) was accomplished via HPLC–ESI–MS/MS experiments on tryptic digests of latent *cg*AUS (sample 5, sequence coverage: 85 %, 441/517 amino acids) and proteolytically activated *cg*AUS (sample 1, sequence coverage: 77 %, 272/350 amino acids). The lists of the found peptides are presented in Online Resource Table S2.

Only three unique peptides of the *cg*AUS2 sequence (A0A075BWS7) were found in the tryptic digest of a solution of sample 1. In the case of the latent sample 5 only one unique peptide of *cg*AUS2 was found. Some peptides of the C-terminal domain of *cg*AUS1 were found in the active enzyme sample. This indicates that the active *cg*AUS1 sample 1 contains trace amounts of latent enzyme. *cg*AUS1 was found to be phosphorylated (Δmass: −2.78 ppm, XCorr: 6.26, charge: 3) or sulfated (Δmass: −3.56 ppm, XCorr: 6.45, charge: 3) at Tyr^230^. This phosphorylation/sulfation was also found in the latent sample 5. Whether this modification is really present in the latent form or the observation is caused by trace amounts of active *cg*AUS in the sample (compare Fig. 3a, b) remains unclear.

### Kinetic properties of active aurone synthase

Kinetic analysis of aurone synthase was performed on a variety of substrates listed in Table 3 and included the two 6′-deoxychalcones butein and marein, the 4-deoxyaurone sulfuretin, the flavonol fisetin, and four common PPO substrates. No differences in activity and substrate specificity were observed between the described active *cg*AUS forms (samples 1–4, identified by HPLC–ESI–MS/MS and ESI–QTOF–MS experiments as *cg*AUS1). The kinetic properties of active *cg*AUS1 were determined using sample 1. To assess substrate specificity of *cg*AUS1 and a putative specific involvement in aurone formation, purified *vv*CO was used as a reference enzyme for a common catechol oxidase.

**Table 3:** Kinetic parameters of *cg*AUS1 and *vv*CO

Substrate	*λ* (nm)	Δ*ε* (l mol^−1^ cm^−1^)	*K*_m_ (mm)	*V*_max_ (µmol min^−1^ mg^−1^)	*k*_cat_ (s^−1^)	*k*_cat_/*K*_m_ (mm s^−1^)
Aurone synthase (*Coreopsis grandiflora*)						
Butein	415	9,320^a^	0.052 ± 0.003	489 ± 17	464 ± 16	8,927 ± 649
Marein	425	13,559^b^	–^c^	–^c^	–^c^	1,647 ± 62^c^
Sulfuretin	475	–^d^	–^d^	–^d^	–^d^	–^d^
Fisetin	280	5,345^e^	–^c^	–^c^	–^c^	2,130 ± 39^c^
4-*tert*-*B*utylcatechol	400	1,150^f^	0.504 ± 0.020	2,256 ± 23	1,563 ± 16	3,101 ± 128
Chlorogenic acid	400	2,566^g^	0.984 ± 0.080	2,549 ± 85	1,782 ± 63	1,811 ± 160
4-Methylcatechol	400	1,350^f^	3.11 ± 0.12	2,299 ± 39	1,593 ± 27	512 ± 22
Catechol	390	1,450^f^	4.04 ± 0.69	1,255 ± 59	869 ± 41	215 ± 38
Catechol oxidase (*Vitis vinifera*)						
Butein	415	9,320^a^	–^c^	–^c^	–^c^	1,300 ± 32^c^
Marein	425	13,559^b^	–^c^	–^c^	–^c^	472 ± 8^c^
Sulfuretin	475	–^d^	–^d^	–^d^	–^d^	–^d^
Fisetin	280	5,345^e^	–^c^	–^c^	–^c^	1,902 ± 35^c^
4-*tert*-Butylcatechol	400	1,150^f^	2.43 ± 0.19	4,110 ± 110	2,847 ± 76	1,170 ± 98
Chlorogenic acid	400	2,566^g^	2.00 ± 0.18	2,274 ± 86	1,575 ± 59	786 ± 76
4-Methylcatechol	400	1,350^f^	7.10 ± 0.12	5,320 ± 33	3,685 ± 22	519 ± 9
Catechol	390	1,450^f^	45.88 ± 1.67	2,857 ± 41	1,979 ± 28	43.1 ± 1.7

^a^Extinction coefficient was determined by calculating difference spectra of sulfuretin and butein

^b^Extinction coefficient was determined at the maximum increase of absorbance by assuming 100 % product formation

^c^A pseudo first-order kinetic model ([S] ≪ *K*_m_) was used to calculate the ratio *k*_cat_/*K*_m_ due to low solubility of the substrates

^d^Extinction coefficient could not be determined due to complex and unspecific product formation

^e^(Jimenez et al. 1998)

^f^(Waite 1976)

^g^(Muñoz et al. 2007)

**Fig. 6:**
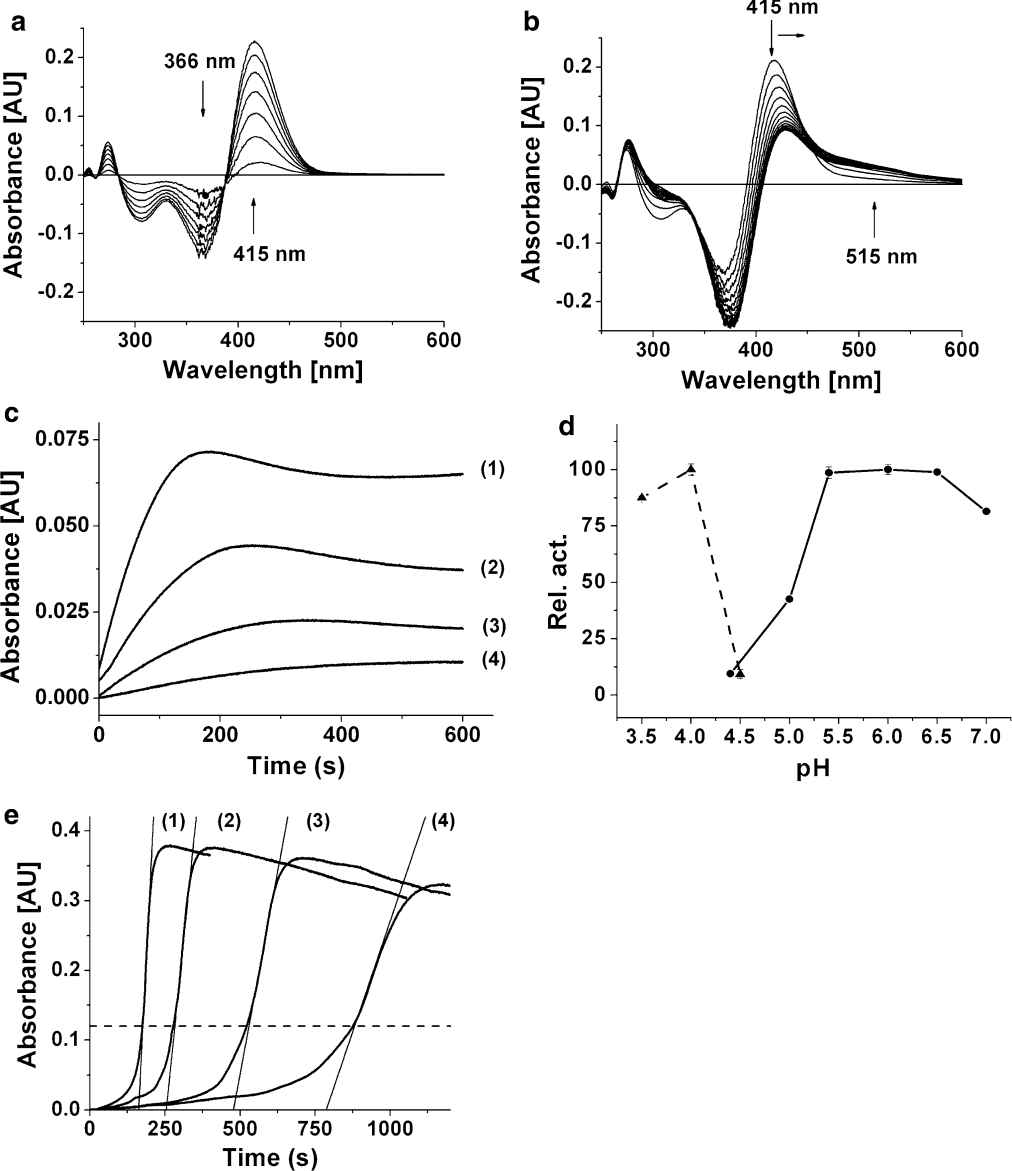
Enzymatic properties of latent and active *cg*AUS1. **a**, **b** Difference spectra of the enzymatic oxidation of butein by *cg*AUS1. The reaction medium contained 25 µm butein in 125 mm sodium citrate buffer, pH 5.5. The final concentration of active *cg*AUS1 was 0.9 nm. Spectra were recorded every 2 min. **a** Oxidative conversion of butein to sulfuretin (0–12 min). **b** Subsequent oxidation of sulfuretin (compare Fig. 8). **c** Time course of the oxidation of sulfuretin by *cg*AUS1 monitored at 475 nm indicating suicide inactivation. The reaction medium contained 50 µm sulfuretin in 125 mm sodium citrate buffer, pH 5.5. The final concentrations of active *cg*AUS1 were 1.2 nm (*1*), 0.6 nm (*2*), 0.3 nm (*3*) and 0.15 nm (*4*). **d** pH optimum of recombinant pro-*cg*AUS1 (*filled triangle*, *dashed lines*) and active *cg*AUS1 (*filled circle*, *solid lines*). 25 µm Butein in 125 mm sodium citrate buffers was used over a range from pH 3.5 to 7.4. **e** Allosteric activation of latent recombinant *cg*AUS1 monitored at 282 nm. The reaction medium contained 50 µm fisetin in 125 mm sodium citrate buffer, pH 5.5. The final concentrations of latent recombinant *cg*AUS1 were 7.2 nm (*1*), 3.6 nm (*2*), 1.8 nm (*3*) and 0.9 nm (*4*). The *dashed line* depicts the beginning of the steady-state region. The absorbance of 120 mAU corresponds to a product concentration of 23 µm

**Fig. 7:**
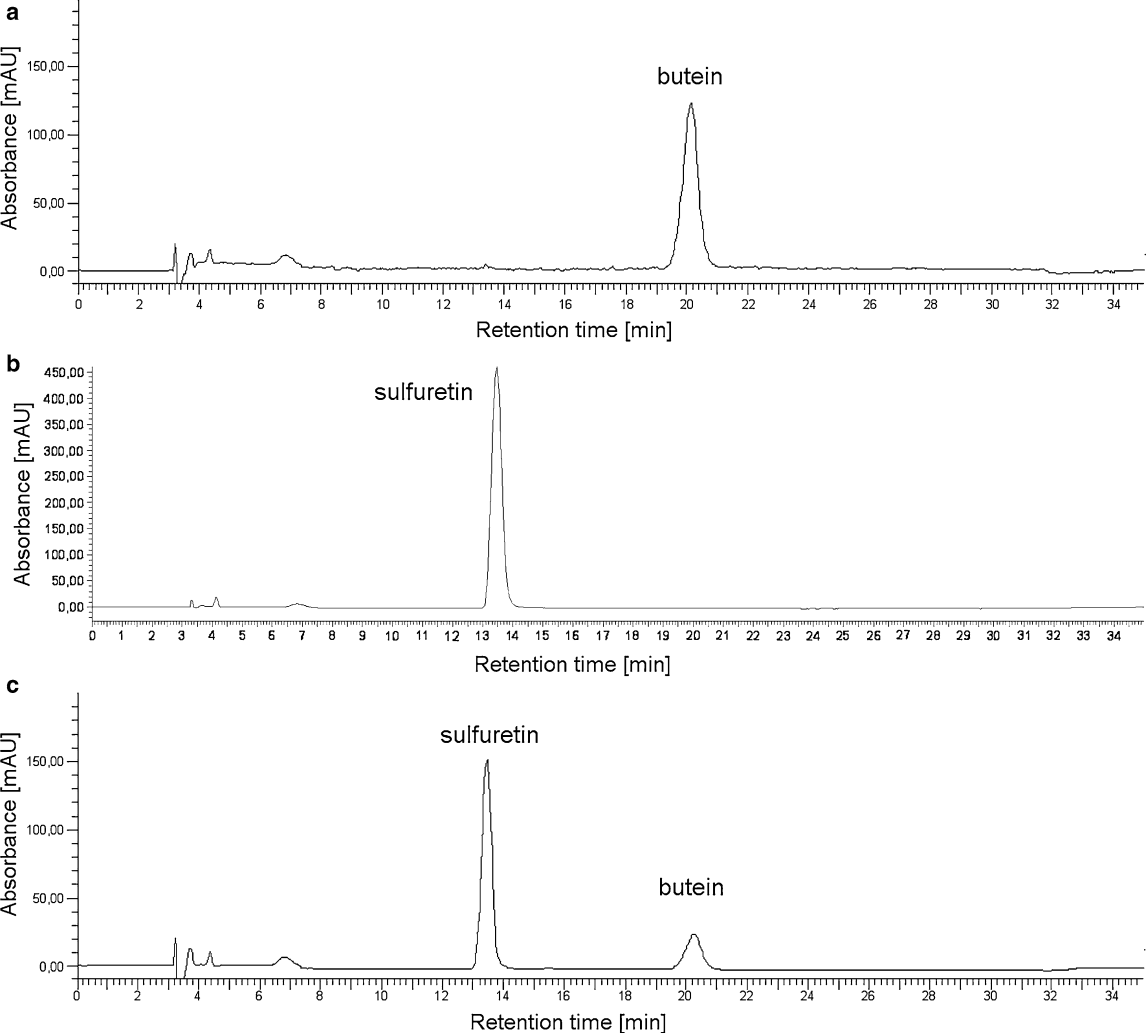
HPLC chromatogramms of the chalcone butein, the aurone sulfuretin and after incubation with active *cg*AUS1. **a** Butein without incubation treatment. **b** Sulfuretin without incubation treatment. **c** Butein incubated with *cg*AUS1

Two subsequent reactions, catalyzed by *cg*AUS1, were spectrophotometrically observed when butein was used as a substrate (Fig. 6a, b). The first reaction (Fig. 6a) represents the oxidation of butein to sulfuretin and causes a maximal increase in absorbance at 415 nm. This assumption was verified by calculating difference spectra of commercially available butein and sulfuretin (Δ*λ*_max_ = 415 nm) as well as analysis of the reaction products by means of RP-HPLC (Fig. 7). The following mechanism for this reaction has been proposed for *Am*AS1: butein is enzymatically oxidized to its chalcone quinone, followed by an oxidative cyclization yielding the aurone sulfuretin which is thought to take place non-enzymatically (Nakayama et al. 2001).

The second reaction (Fig. 6b) was observed only after the majority of butein had been oxidized to sulfuretin. This was visible by a decrease in absorbance at 415 nm attended by a shift to higher wavelengths and a newly occurring broad absorption band from 480–600 nm. Sulfuretin is an *o*-diphenol and therefore a potential substrate for PPOs. The time course of the oxidation of sulfuretin (Δ*λ*_max_ = 475 nm) showed a nonlinear behavior with a slope decreasing over time even reaching an idle state, although only a minority of substrate has been oxidized (Fig. 6c). The final quantity of product formation strongly depended on the amount of enzyme used for the assay (Fig. 6c). The observed decrease in absorbance is most likely a consequence of polymerization of the oxidation products indicated by the broad absorption band around 520 nm and also indicated by HPLC analysis from assays with high amounts of protein, in which neither products nor substrates could be observed.

In contrast to *Am*AS1, no hydroxylation reaction was detected when isoliquiritigenin was used as a substrate, even when hydrogen peroxide was added to the reaction medium to omit the lag phase by transferring the copper center of the protein to the reactive *oxy*-form (reviewed by Ramsden and Riley 2014). In addition, no introduction of a third hydroxyl group in the B-ring of the substrates butein and marein could be observed by means of HPLC analysis.

The pH optimum of *cg*AUS1 is between pH 5.4–6.5 for butein (Fig. 6d). Due to the higher stability of *o*-diphenolic substrates (as well as the resulting *o*-quinones) at acidic pH values, kinetic parameters for *cg*AUS1 as well as for *vv*CO were determined at pH 5.5. Marein and fisetin showed a low affinity to *cg*AUS1 and the expected *K*_*m*_ values were significant higher than the solubility of these substrates (up to 75 µm). This prevented an accurate estimation of *K*_*m*_ by nonlinear regression. Instead *k*_cat_/*K*_m_ values were determined by linear fitting to pseudo first-order kinetics ([S] ≪ *K*_m_). This was also the case for butein, marein and fisetin with *vv*CO enzyme. *cg*AUS1 displayed a *K*_m_ value of 52 ± 3 µm and also the highest catalytic efficiency towards butein (Table 3). The *k*_cat_/*K*_m_ value of marein is more than five times lower than for butein. *vv*CO instead showed the highest catalytic efficiency towards fisetin and a more than six times lower efficiency towards butein than *cg*AUS1. In general, *cg*AUS1 exhibits lower *K*_m_ values and possesses higher *k*_cat_/*K*_m_ values. 4-Methylcatchol is the only exception showing very similar catalytic efficiencies for both enzymes.

### Kinetic properties of latent aurone synthase

The recombinantly expressed *cg*AUS1 (Kaintz et al. 2014) exhibited identical properties to the latent *cg*AUS1 purified from the natural source. However, the enzyme purified from *Coreopsis* petals contained low amounts of active *cg*AUS1. Recombinantly expressed *cg*AUS1 was therefore used to describe the characteristics of the latent enzyme.

The pH optimum of latent *cg*AUS1 reflects the acidic activation of the pro-enzyme in a very narrow range around pH 4.0 (Fig. 6d). An allosteric activation was observed when fisetin was used at pH 5.5 as a substrate similar to that described for latent *vv*CO (Valero and Garcia-Carmona 1992). The lag period is dependent on the enzyme concentration but the steady-state rate is reached at a distinct product concentration (Fig. 6e). An in situ product concentration of approximately 23 µm is determined to be the critical concentration in the case of fisetin. At this concentration of oxidized fisetin *cg*AUS1 is fully activated. The experiments also show that a potential influence of the incubation time of the enzyme with oxidized fisetin is negligible under the chosen conditions. Allosteric activation was observed for all substrates tested, except for chalcones where no activiation occurred. Instead, *cg*AUS1 showed a steady-state reaction rate, but only a few percent of its maximum activity at pH 4.0 during the complete reaction time (comp. Figure 6d).

## Discussion

### Purification and identification of aurone synthase

Polyphenols and their corresponding quinones are highly reactive compounds that form polymerized products. These are responsible for protein cross-linking and cause protein precipitation (Mcmanus et al. 1981; Ito et al. 1984; McDowell et al. 1999; Kumar et al. 2000). It is absolutely essential therefore, to remove these compounds from crude extracts which serve as a source for a pure protein. ATPS is a commonly used first step in protein isolation/purification (reviewed by Aguilar and Rito-Palomares 2010 and Hong Yang 2013). In this study ATPS systems based on Triton X-114 and PEG (Sojo et al. 1998) were combined to remove pigments and polyphenols which accumulate in the more hydrophobic detergent-rich phases. The successful combination of Triton X-114 ATPS, ammonium sulfate precipitation and PEG-4000 ATPS is a decisive improvement in the protein purification procedure from polyphenol rich natural sources.

The chromatograms, especially at a small scale, displayed good resolutions of differing protein peaks. AUS activity, however, could be determined over a wide range of the gradient. This indicates that *cg*AUS might contain diverse modifications, resulting in overlapping peaks in the chromatograms. Cation and anion exchange chromatography were therefore combined to benefit from different titration curves of the different forms. From the large number of AUS peaks in the obtained chromatograms, those containing high activity and high amounts of *cg*AUS were chosen for further purification and investigation.

Despite the diversity and high number of active *cg*AUS forms observed during purification, only one latent and two partially proteolytically cleaved forms of lower quantity were observed during purification. Mass spectrometric analyses revealed that all the active *cg*AUS forms were caused by unspecific proteolytic cleavage of *cg*AUS1 in combination with a phosphorylation or sulfation. Recent works also reported an unspecific C-terminal proteolytic cleavage of PPOs resulting in several heterogeneous forms (Mauracher et al. 2014; Zekiri et al. 2014). Only a small number of unique peptides from *cg*AUS2 were found. The abundance and prevalence of *cg*AUS1 in petals of *C. grandiflora* is in accordance with reported gene expression studies (Kaintz et al. 2014) which also showed a correlation of *cg*AUS1 expression with aurone accumulation in petals of *C. grandiflora.*

### Unique structural features of *cg*AUS1, a member of the novel group 2 PPOs

*cg*AUS1 shows the highest sequence identity for the catalytically active domains (53–65 %, excluding the transit peptide and the C-terminal domain) with PPOs from dandelion (*Taraxacum officinale*) and a considerably lower sequence identity to other PPOs (40–50 %). It was suggested recently, that dandelion PPOs are clustered into two distinct groups (Dirks-Hofmeister et al. 2014). The grouping of these PPOs correlates with differences at the CuB binding site, namely at the amino acid directly following the second copper-binding histidine (H_B2_ + 1) (Dirks-Hofmeister et al. 2014). This residue is a bulky, charged arginine in group 2 PPOs and a small uncharged residue in group 1 PPOs. Notably, the selected PPOs exhibiting monophenolase activity share small and hydrophobic residues in this position (Fig. 5b). Crystal structures of tyrosinase from *Bacillus megaterium* demonstrated the importance of residue H_B2_ + 1 for docking of substrates to the active site (Goldfeder et al. 2014) recently. Due to the absence of a charged residue in this position an alternative substrate docking for plant tyrosinases seems to be very likely.

Phylogenetic analysis shows that *cg*AUS1 clusters in the group 2 PPOs from *T. officinale* (Fig. 5c, the corresponding alignment is presented in Online Resource Fig. S3). Therefore we propose that these PPOs form a novel and, with exception of recombinantly expressed pro-PPOs from *T. officinale*, previously uncharacterized subclass of plant PPOs. The sequence alignment of group 1 and group 2 PPOs expose a novel characteristic feature of the group 2 PPOs (Fig. 5b). There exists an insertion just before the CuB binding site (Fig. 5b, e.g. V^237^ANG^240^ in the *cg*AUS1 sequence). Due to its location in a loop region on the surface and near to the active site of catechol oxidases (pdb-entries: 1bt3, 2p3x) it might influence substrate docking to the active site. This might also explain the more specific substrate binding of *cg*AUS1 as *cg*AUS1 displays generally lower *K*_*m*_ values and, with exception of fisetin, a higher catalytic efficiency than *vv*CO (Table 3).

Indications of a putative phosphorylated or sulfated residue of active *cg*AUS1 forms were obtained by ESI–QTOF–MS experiments. The residue Tyr^230^ was found to be phosphorylated or sulfated by means of HPLC–ESI–MS/MS experiments of tryptic digested active *cg*AUS1 with a high degree of confidence (Online Resource Table S2). To date, only one PPO from dormant terminal buds in poplar (*Populus simonii* × *P. nigra*) was found to be phosphorylated (Liu et al. 2011). The phosphorylation in poplar PPO, however, was found in the shielding C-terminal domain and not in the main core. Notably, the modification in *cg*AUS1 was found in the immediate vicinity of the loop extension (Fig. 5a). Its role remains unclear at this stage of the work.

The results of analytical SDS-PAGE for the purified latent *cg*AUS forms differ under reducing and non-reducing conditions. Consequently, we concluded that the C-terminal domain, shielding the active site of the protein, is connected to the main core of the enzyme by a disulfide bond. This hypothesis was verified by ESI–QTOF–MS measurements of the active *cg*AUS forms (sample 1 to sample 4) under reducing and non-reducing conditions. The residual C-terminal peptide of active *cg*AUS starts a few amino acids behind the KFDV-motif, which is highly conserved in plant PPOs (Fig. 5d). We assume, that the main core cysteine Cys^206^ is involved in disulfide linkage, as the highly conserved Cys^334^ is not accessible (comp. pdb-entry 2p3x). An intermolecular disulfide linkage has been reported to be responsible for the tetramerization of recombinant expressed PPO-6 and PPO-7 from *T* *. officinale* (Dirks-Hofmeister et al. 2012, 2014) and Cys^197^ was identified to be involved in the tetramerization. An intramolecular crosslink between the catalytically active main core and the shielding C-terminal domain has not been reported so far and one function might be that it stabilizes the interface between the two domains. The pro-enzyme has to be cleaved at three different positions to result in the active form (Fig. 5a, cleavage sites are indicated by violet lines). An involvement of the remaining C-terminal peptide (D^438^GVFTTPCDPEYAGG^452^) in the catalytic activity of the enzyme is very unlikely, because the peptide is short (7 amino acids upstream and downstream of Cys^445^, respectively), and the Cys^206^ is located in a helix further away of the active site.

### Kinetic characterization suggests alternative 4-deoxyaurone formation in Asteraceae species

In the assays with purified *cg*AUS1, the oxidation of butein to sulfuretin followed the route described for *Am*AS1 (Fig. 6a). Additionally, the oxidation of sulfuretin to the corresponding *o*-quinone could be observed as well (Fig. 6b). However, this was strongly dependent on the presence of large enzyme amounts in the assay (Fig. 6c) and can be explained by the suicide inactivation of PPOs (Escribano et al. 1989; Chazarra et al. 1997; Garcia-Molina et al. 2005; Land et al. 2008; Muñoz-Muñoz et al. 2008, 2011, 2012a, b; Ramsden et al. 2009; Ramsden and Riley 2010a). It has been reported that *Am*AS1 shows virtually no oxidation of aurones (Nakayama et al. 2000) and comparison with our results suggests that *Am*AS1 might exhibit suicide inactivation towards aurones. In accordance to literature (Chazarra et al. 1997; Ramsden and Riley 2010b), the suicide substrates 4-*tert*-butylcatechol and 4-methylcatechol were identified to inactivate *cg*AUS1. As expected, *vv*CO demonstrated irreversible inactivation towards *p*-substituted substrates as well.

**Fig. 8:**
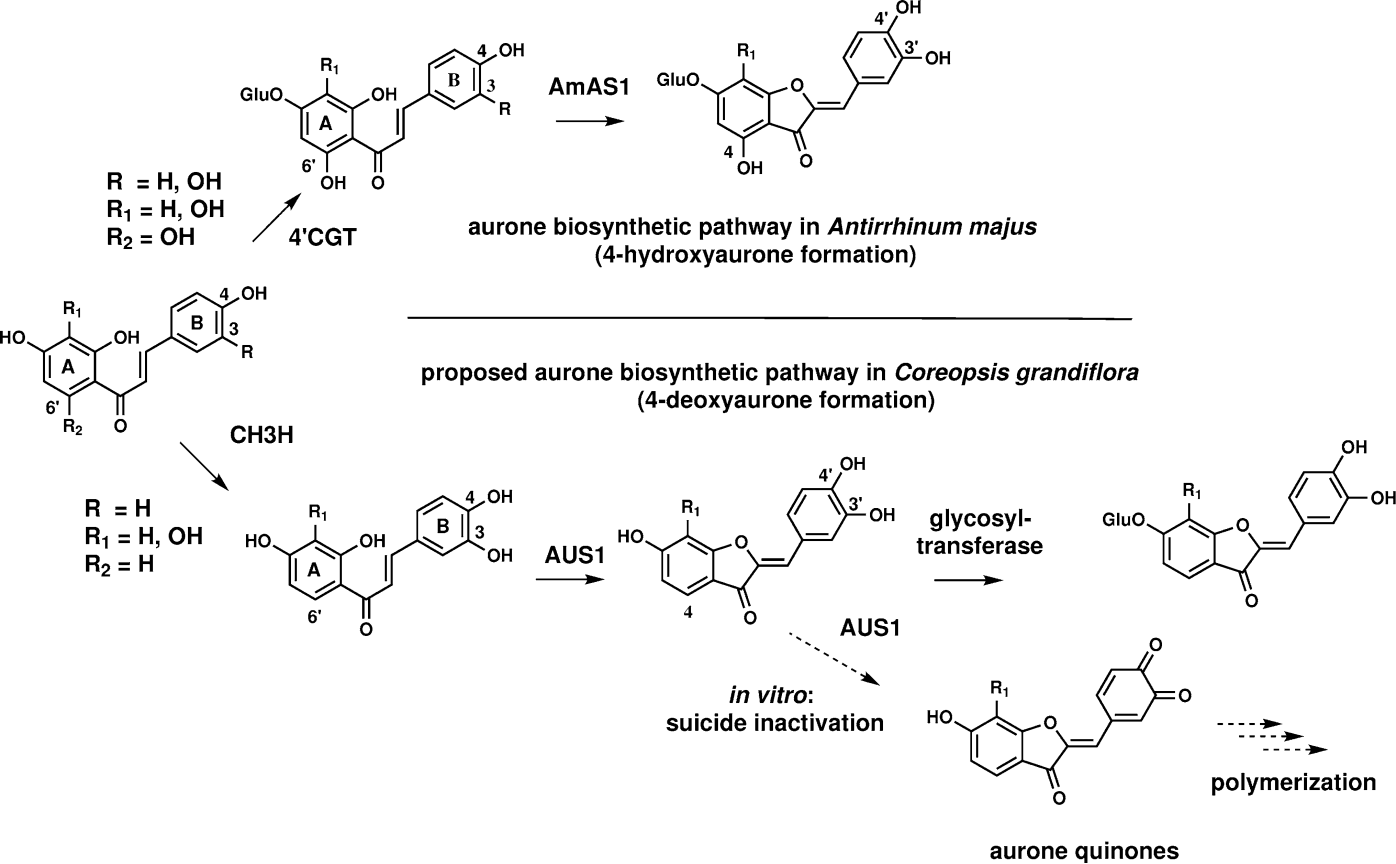
4-Hydroxyaurone and proposed 4-deoxyaurone biosynthetic pathway. The *upper part* shows the aurone biosynthetic pathway in *Antirrhinum majus* (4-hydroxyaurone formation). Chalcones are glycosylated by a chalcone 4′-*O*-glucosyltransferase (4′CGT) and transported to the vacuole. Aureusidin synthase (*Am*AS1) possesses monophenolase and diphenolase activity. The *lower figure* shows the proposed biosynthetic pathway in *C. grandiflora* (4-deoxyaurone formation). Chalcones are hydroxylated by a chalcone 3-hydroxylase (CH3H); aurone synthase (*cg*AUS1) possesses only diphenolase activity; aurones are glycosylated by a glycosyltransferase. Oxidation of aurones resulted in suicide inactivated enzyme in vitro

Kinetic parameters demonstrate a high specificity and efficiency of *cg*AUS1 for butein in comparison to *vv*CO. As butein is a naturally occurring substrate in *Coreopsis species* (Crawford and Smith 1983), this supports the assumption that *cg*AUS1 is specifically involved in the aurone biosynthesis. The significant higher affinity of butein to *cg*AUS1 than to *vv*CO indicates that specific residues of *cg*AUS1 are involved in the coordination of butein to its active site. Confirmation and identification of potential residues are, however, subject of analysis of crystal structures of active *cg*AUS1. In contrast to the determination of kinetic parameters of *cg*AUS1 towards butein (nonlinear regression), the *k*_cat_/*K*_m_ value of *cg*AUS1 towards marein had to be determined by linear fitting to pseudo first-order kinetics ([S] ≪ *K*_m_), although both substrates (butein and marein) were applied at the same concentration ranges (10–75 µm). This indicates a significantly lower affinity of *cg*AUS1 to marein. Unfortunately, to date, no chalcone glycoside/chalcone aglycone pair is commercial available, therefore it remains unclear whether this difference in affinity is caused by the glycosyl group of marein or by the hydroxyl group at position 3′. However, the occurence of chalcone aglycones (butein, okanin, lanceoletin) and the corresponding aurone aglycones (sulfuretin, maritimetin, leptosidin) in *Coreopsis* (comp. Fig. 1) combined with the observed lower affinity of marein strongly support that 4-deoxyaurone formation in *C. grandiflora* occurs at the level of chalcone aglycones possessing two vicinal hydroxyl groups in the *B*-ring. This hypothesis is furthermore supported by the reported significantly higher affinity of a glycosyltransferase from *C. grandiflora* for sulfuretin than for butein (Halbwirth et al. 1997), indicating that glycosylation occurs on the level of aurones. Combination with previous studies on 4-deoxyaurone formation in *Bidens ferulifolia* (Miosic et al. 2013) strongly suggests that an alternative pathway for aurone biosynthesis exists in Asteraceae species, in addition to that proposed for *A. majus* (Ono et al. 2006a) (Fig. 8). The predicted localization in chloroplasts (or chromoplasts) of *cg*AUS1 (Kaintz et al. 2014) does not contradict its involvement in aurone biosynthesis. Recently, Araji et al. (2014) proposed a novel involvement of PPOs, most likely localized in chloroplasts, in the secondary metabolism of walnut (*Juglans regia*) leaves. The authors suggested that the substrates, biosynthesized in the cytoplasm, would presumably need to be transported back to the chloroplast for catalysis by PPO. However, to date no information of prerequisite transport mechanisms are available.

### Allosteric activation: latent *cg*AUS1 is fully activated at a distinct *o*-quinone concentration

Although allosteric activation of latent PPOs has been reported some time ago and several latent PPOs are recombinantly available, the phenomenon is rarely described and still poorly understood. Allosteric activation of *cg*AUS1 occurs at neutral or slightly acidic pH ranges. This behavior has also been reported for other latent PPOs (Valero and Garcia-Carmona 1992, 1998; Nillius et al. 2008; Dirks-Hofmeister et al. 2012, 2014). Previous studies reported that the lag period remained constant with varying the enzyme concentration (Valero and Garcia-Carmona 1992). In the case of latent *cg*AUS1, however, the lag period strongly depended on enzyme concentration and the steady state was reached at a defined product concentration (Fig. 6e). The absence of allosteric activation during the oxidation of chalcones suggests that the activation of latent *cg*AUS1 is caused by *o*-quinones, as aurones themselves do not possess a quinoide structure. The quinone-binding site might be within the interface of the shielding C-terminal domain and the main core, which might result in a similar structural rearrangement as proposed for an acidic activation or activation with SDS. The interaction is most likely irreversible and covalent, because the *o*-quinones are known to be highly reactive and likely to undergo rapid polymerization reactions and are also responsible for protein cross-linking (Ito et al. 1984; Burzio and Waite 2000; Rollett et al. 2013). The existence of a concentration threshold of *o*-quinones where the enzyme is fully activated is a novel finding. However, the principle behind this behavior remains unclear and may vary, depending on the reactivity of the *o*-quinone. We propose that allosteric activation can be a general feature of pro-PPOs and may trigger important physiological defense mechanisms, for example, those induced by tissue damage or during oxidative stress in plants and also in fungi.

## Conclusion

The purified *cg*AUS was identified as *cg*AUS1 and the corresponding cDNA clone was reported to be particularly expressed in petals recently. Our results reveal that aurone synthase is a member of the novel group 2 PPOs and that an insertion in a loop region near to the active site, which might be involved in substrate docking, is characteristic for this group. A phosphorylation/sulfation of unknown function was found in the immediate vicinity of the loop extension. The disulfide crosslink of the C-terminal domain to the main core is a novel structural feature of plant PPOs. Kinetic characterization of *cg*AUS1 suggests that aurone formation might occur at the stage of chalcone agylcones which would constitute an alternative aurone biosynthetic pathway in Asteraceae species in comparison to that described for *A. majus* (Plantaginaceae). The availability of large amounts of highly purified and characterized aurone synthase enables crystallization experiments. Structural analysis of latent and active *cg*AUS1 will provide further insights to complement the presented novel findings.

### *Author contribution*

CM.: Planned experiments; Performed experiments; Analyzed data; Wrote the paper; S.G.M.: Planned experiments; Analyzed data; S.P.: Performed experiments; R.L.M.: Performed experiments; Analyzed data; H.H.: Contributed reagents or other essential material; Performed experiments; Analyzed data; other; A.R.: Planned experiments; Contributed reagents or other essential material; other.

## Electronic supplementary material

Below is the link to the electronic supplementary material.

Supplementary material 1 (PDF 442 kb)

Supplementary material 2 (PDF 422 kb)

Supplementary material 3 (PDF 1342 kb)

Supplementary material 4 (PDF 35 kb)

Supplementary material 5 (PDF 21 kb)

## Abbreviations

AEX: Anion exchange
ATPS: Aqueous two-phase separation
*cg*AUS: *Coreopsis grandiflora* aurone synthase
CEX: Cation exchange
CV: Column volumes
DTT: dl-Dithiothreitol
MWCO: Molecular weight cut off
PPO(s): Polyphenol oxidase(s)
*vv*CO: *Vitis vinifera* catechol oxidase
