# Supplementary material for: Latent and active aurone synthase from petals of C. grandiflora: a polyphenol oxidase with unique characteristics
Source: Planta. 2015 Feb 20;242(3):519–37. doi: 10.1007/s00425-015-2261-0 (PMC4540782; doi:10.1007/s00425-015-2261-0)

ONLINE RESOURCE

Latent and active aurone synthase from petals of *C. grandiflora*: a polyphenol oxidase with unique characteristics

Christian Molitor, Stephan Gerhard Mauracher, Sanela Pargan, Rupert L. Mayer, Heidi Halbwirth, Annette Rompel*

* Corresponding author: Annette Rompel, Institut für Biophysikalische Chemie, Fakultät für Chemie, Universität Wien, Althanstraße 14, 1090 Wien, Austria.
E-mail: annette.rompel@univie.ac.at

Online Resource Fig. S1 Purification, yield and SDS-PAGE of catechol oxidase from *Vitis vinifera* (*vv*CO). Catechol oxidase from *Vitis* vinifera (*vv*CO) was purified to homogeneity and used as a reference enzyme to compare the substrate specificity of aurone synthase with a confirmed catechol oxidase.

Isolation of *vv*CO was performed analogous to the procedure described for *cg*AUS, starting form 1.5 kg of grape berries. Enzymatic assays were performed by monitoring the oxidation of 5 mM 4-*tert*-butylcatechol in 1 ml 125 mM sodium citrate pH 5.5 at 400 nm. Two different active forms were observed during purification (*vv*CO 1; *vv*CO 2). A yield of 1.00 mg and 0.47 mg, respectively, was obtained and the purity of both samples was verified by SDS-PAGE (Fig. 3c). The sample containing the higher amount of protein was used for determining kinetic parameters of *Vitis vinifera* catechol oxidase. **a** CEX chromatography using SP-Sepharose FF. Buffer A: 20 mM sodium acetate, pH 5.0. Buffer B: 20 mM NaOAc, pH 5,0, 700 mM NaCl. **b** CEX chromatography using Mono S. Buffer A: 20 mM sodium acetate, pH 5.0. Buffer B: 20 mM NaOAc, pH 5,0, 700 mM NaCl. **c** Polishing CEX chromatography using Mono S. Buffer A: 20 mM sodium acetate, pH 5.0. Buffer B: 20 mM NaOAc, pH 5,0, 700 mM NaCl. **d** Polishing CEX chromatography using Mono S. Buffer A: 20 mM sodium acetate, pH 5.0. Buffer B: 20 mM NaOAc, pH 5,0, 700 mM NaCl. **e** SDS-PAGE of purified *vv*CO; Laemmli sample buffer contained DTT. Lane-number 1: *vv*CO 1; lane-number 2: *vv*CO 2; M: molecular weight marker. **f** SDS-PAGE of purified *vv*CO; Leammli sample buffer did not contain DTT. Lane-number 1: *vv*CO 1; lane-number 2: *vv*CO 2; M: molecular weight marker

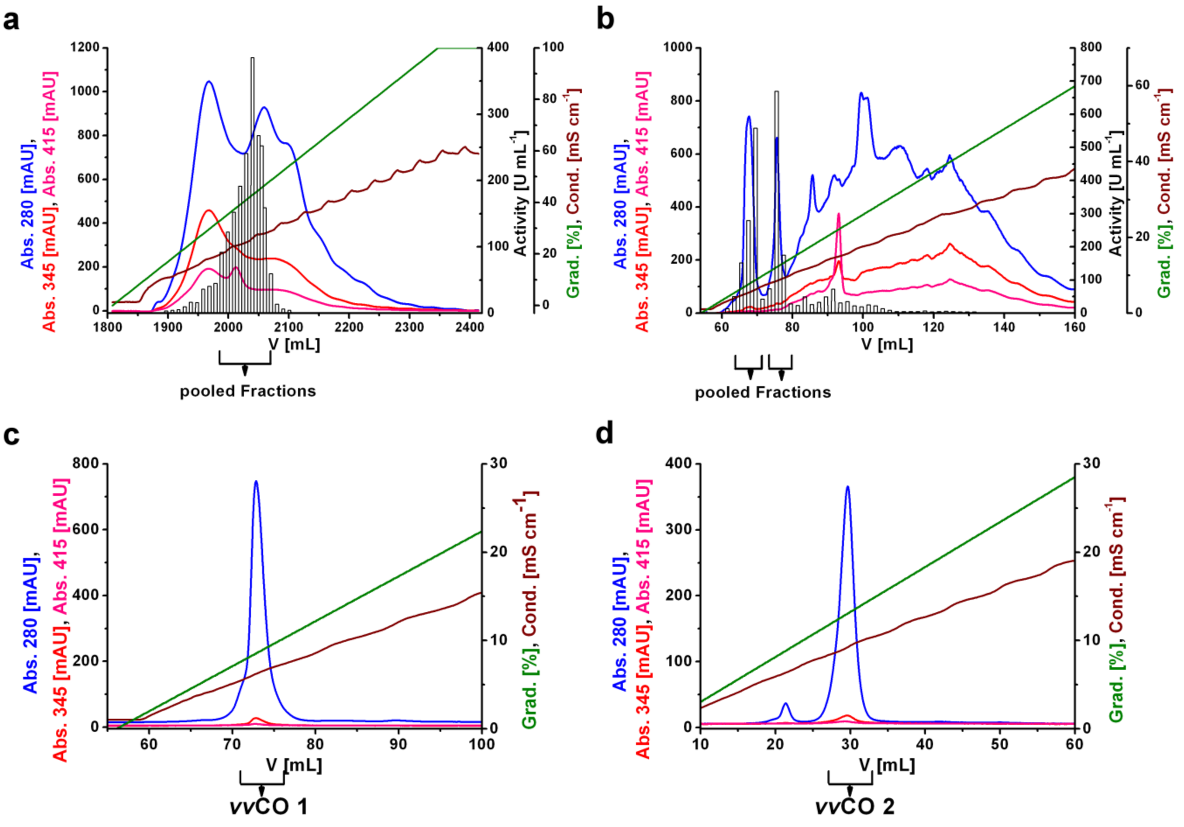

e

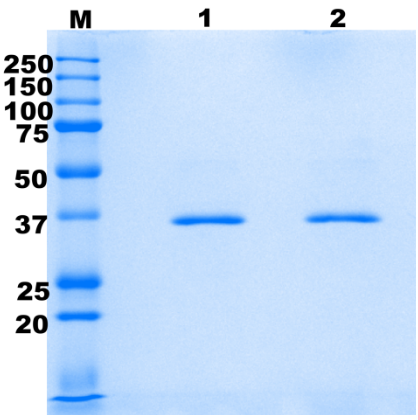

f

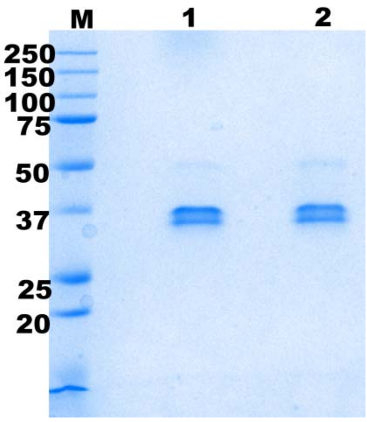

Supplement: Supplementary file 1 — Supplementary material 1 (PDF 442 kb) [file 425_2015_2261_MOESM1_ESM.pdf]
