# Supplementary material for: Latent and active aurone synthase from petals of C. grandiflora: a polyphenol oxidase with unique characteristics
Source: Planta. 2015 Feb 20;242(3):519–37. doi: 10.1007/s00425-015-2261-0 (PMC4540782; doi:10.1007/s00425-015-2261-0)
Supplement: Supplementary file 2 — Supplementary material 2 (PDF 422 kb) [file 425_2015_2261_MOESM2_ESM.pdf]

ONLINE RESOURCE

Latent and active aurone synthase from petals of *C. grandiflora*: a polyphenol oxidase with unique characteristics

Christian Molitor, Stephan Gerhard Mauracher, Sanela Pargan, Rupert L. Mayer, Heidi Halbwirth, Annette Rompel*

* Corresponding author: Annette Rompel, Institut für Biophysikalische Chemie, Fakultät für Chemie, Universität Wien, Althanstraße 14, 1090 Wien, Austria.

E-mail: annette.rompel@univie.ac.at

Online Resource Fig. S2 Positive mode ESI-QTOF mass spectra of purified active *cg*AUS samples. Just prior to measurements acetonitrile and formic acid were added to a final concentration of 25 % (v/v) ACN and 0.05 % (v/v) formic acid. Non-reducing conditions: samples were desalted to 10 mM ammonium acetate pH 5.0. Reducing conditions: samples were incubated for 45 min at 50 °C in 20 mM ammonium acetate pH 7.5, supplemented with 50 mM DTT. Masses determined from charge deconvolution of these mass spectra and the respective deduced sequence of identified mass species are listed in Table S3. **a** Entire and magnified mass spectra of sample 2 under non-reducing conditions. **b** Entire and magnified mass spectra of sample 2 under reducing conditions. **c** Entire and magnified mass spectra of sample 3 under non-reducing conditions. **d** Entire and magnified mass spectra of sample 3 under reducing conditions. **e** Entire and magnified mass spectra of sample 4 under non-reducing conditions. **f** Entire and magnified mass spectra of sample 4 under reducing conditions

a

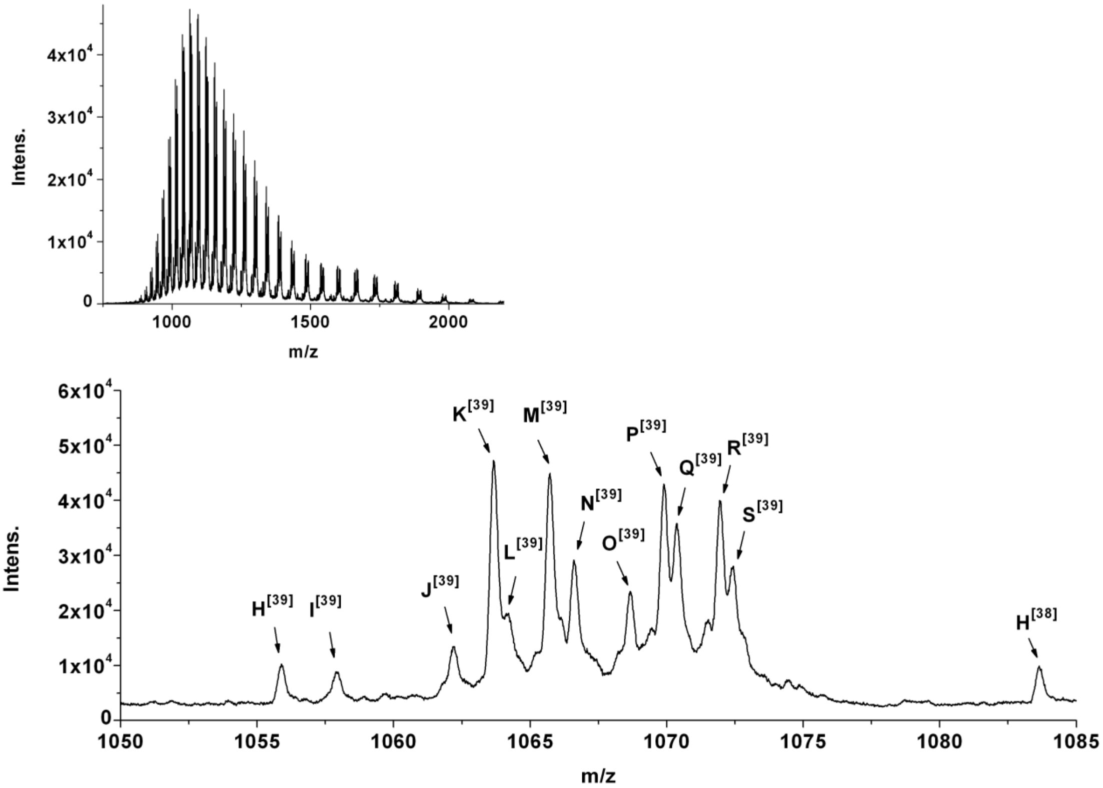

b

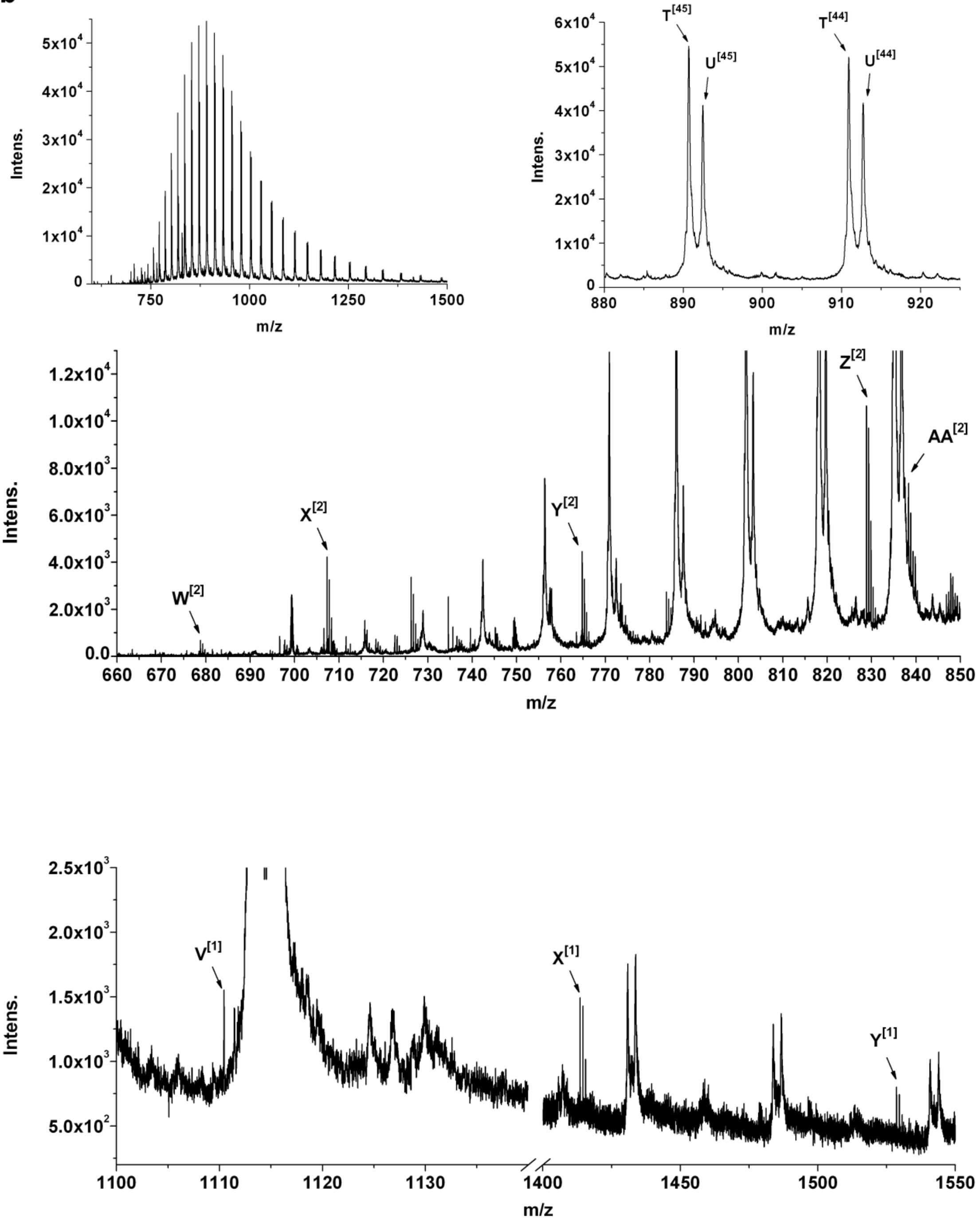

c

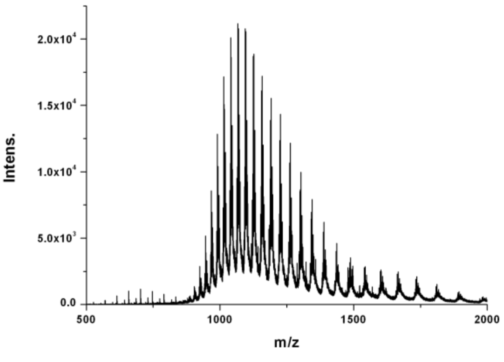

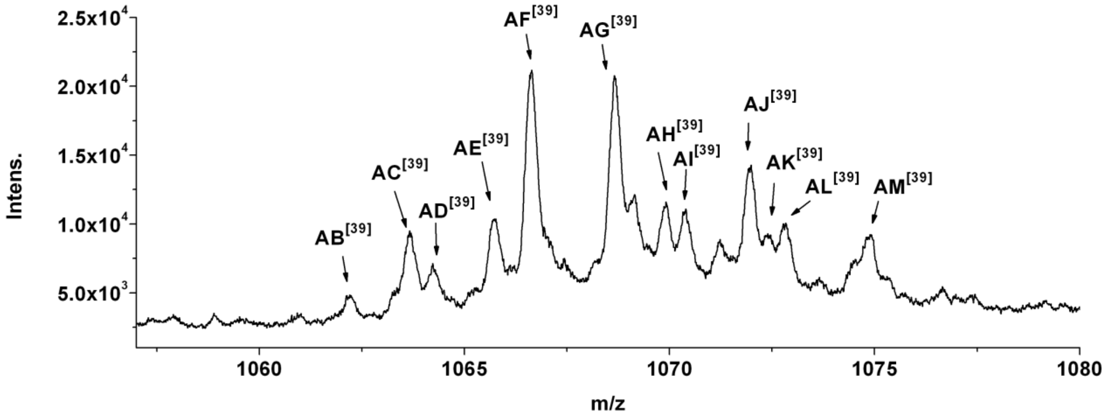
Intens.
m/z
AB[39]
AC[39]
AD[39]
AE[39]
AF[39]
AG[39]
AH[39]
AI[39]
AJ[39]
AK[39]
AL[39]
AM[39]

d

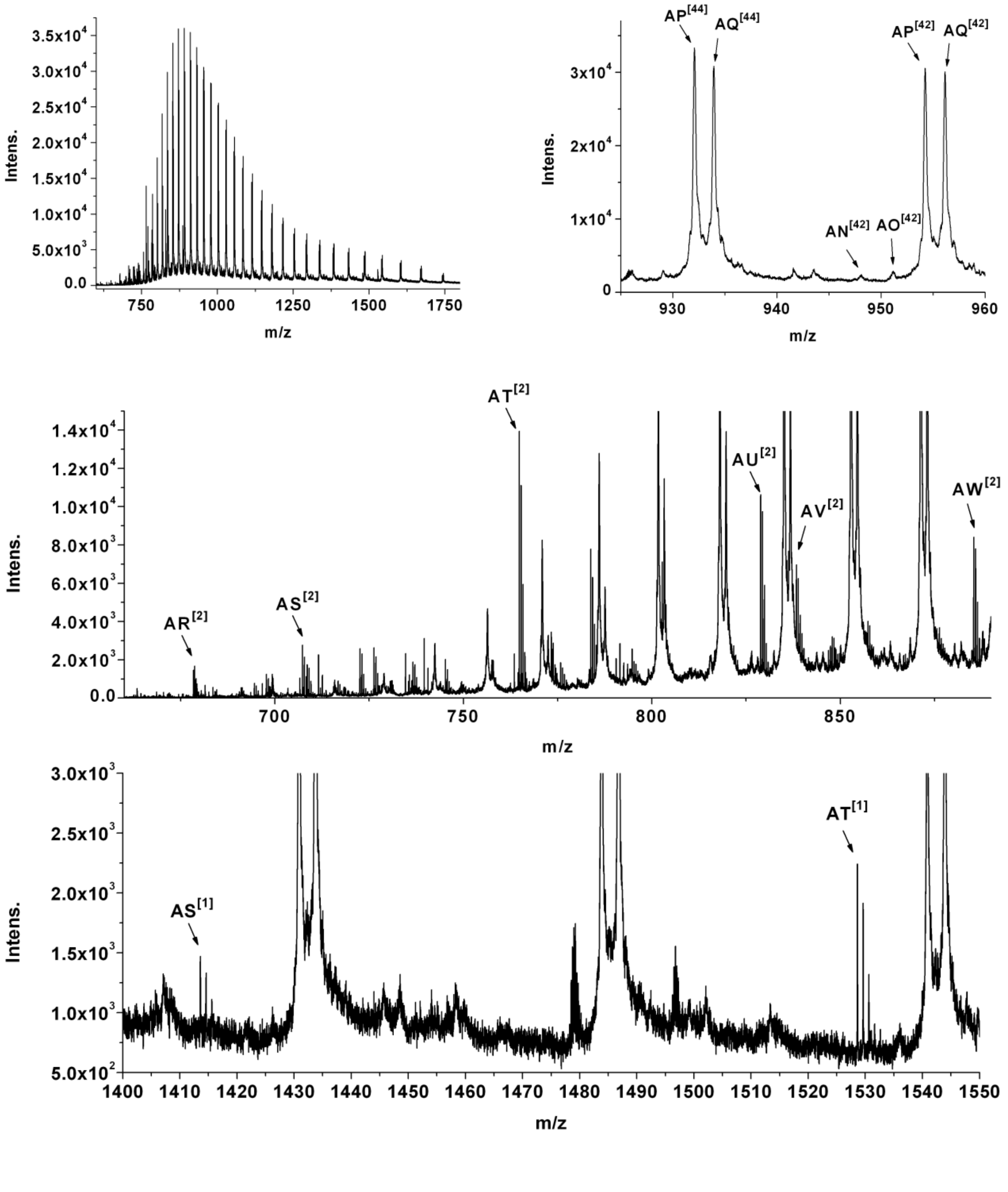
Intens.
m/z
AP[44]
AQ[44]
AN[42]
AO[42]
AP[42]
AQ[42]
AR[2]
AS[2]
AT[2]
AU[2]
AV[2]
AW[2]
AS[1]
AT[1]

e

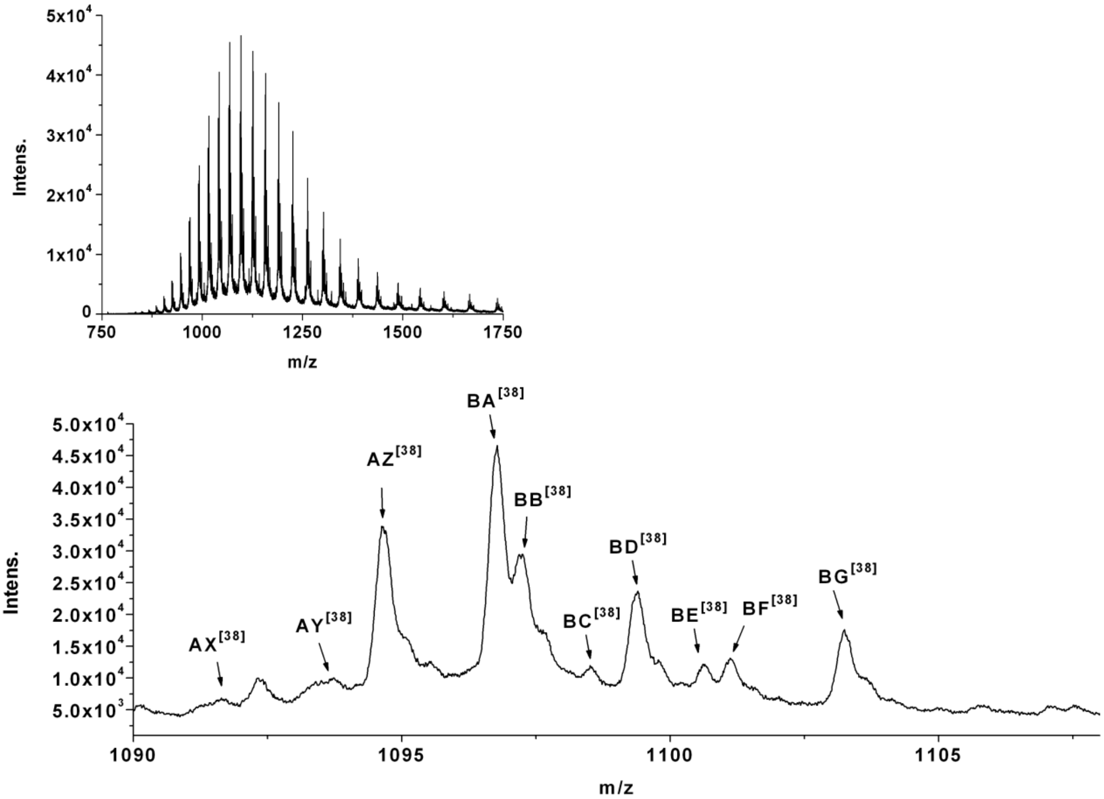
Intens.
m/z
AX[38]
AY[38]
AZ[38]
BA[38]
BB[38]
BC[38]
BD[38]
BE[38]
BF[38]
BG[38]

f

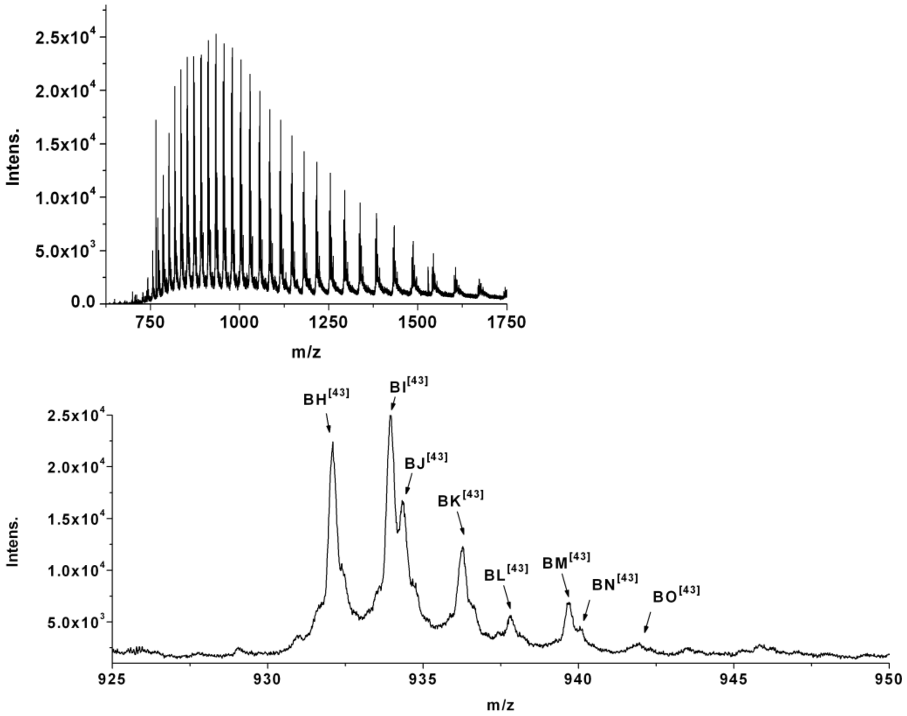
Intens.
m/z
BH[43]
BI[43]
BJ[43]
BK[43]
BL[43]
BM[43]
BN[43]
BO[43]

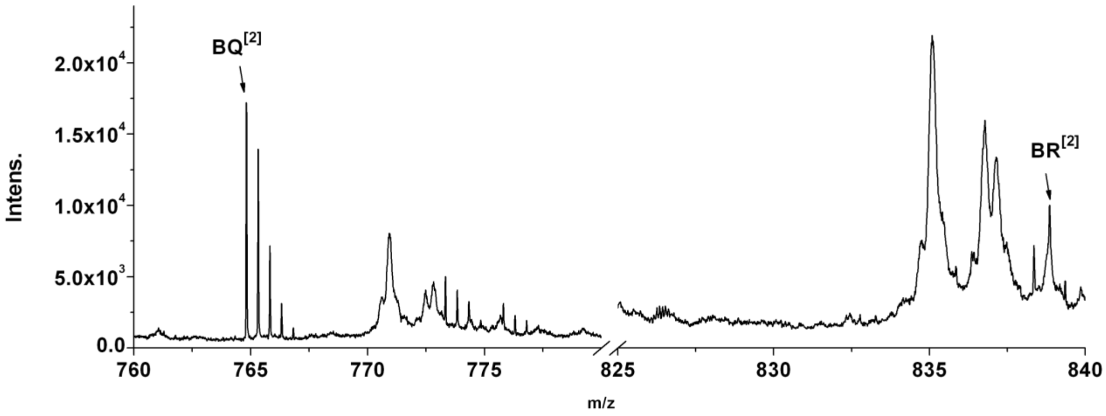
BQ[2]
BR[2]
Intens.
2.0x10^4
1.5x10^4
1.0x10^4
5.0x10^3
0.0
760
765
770
775
825
830
835
840
m/z

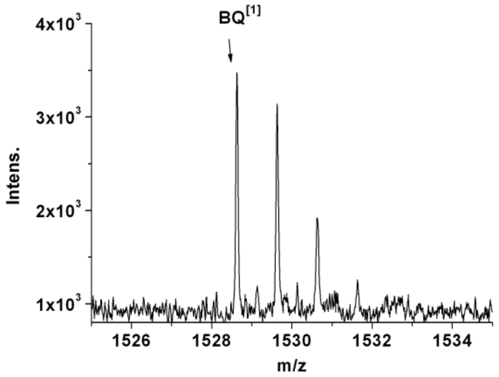
BQ[1]
Intens.
4x10^3
3x10^3
2x10^3
1x10^3
1526
1528
1530
1532
1534
m/z
