# Supplementary material for: Latent and active aurone synthase from petals of C. grandiflora: a polyphenol oxidase with unique characteristics
Source: Planta. 2015 Feb 20;242(3):519–37. doi: 10.1007/s00425-015-2261-0 (PMC4540782; doi:10.1007/s00425-015-2261-0)

ONLINE RESOURCE

Latent and active aurone synthase from petals of *C. grandiflora*: a polyphenol oxidase with unique characteristics

Christian Molitor, Stephan Gerhard Mauracher, Sanela Pargan, Rupert L. Mayer, Heidi Halbwirth, Annette Rompel*

* Corresponding author: Annette Rompel, Institut für Biophysikalische Chemie, Fakultät für Chemie, Universität Wien, Althanstraße 14, 1090 Wien, Austria.
E-mail: annette.rompel@univie.ac.at

Online Resource Fig. S3 Alignment corresponding to the phylogenetic tree (Fig. 5c). The red line indicates the grouping of PPOs in group 1 PPOs and group 2 PPOs (compare phylogenetic tree, Fig. 5c). The characteristic insertion in a loop region near the active site of group 2 PPOs is colored green.

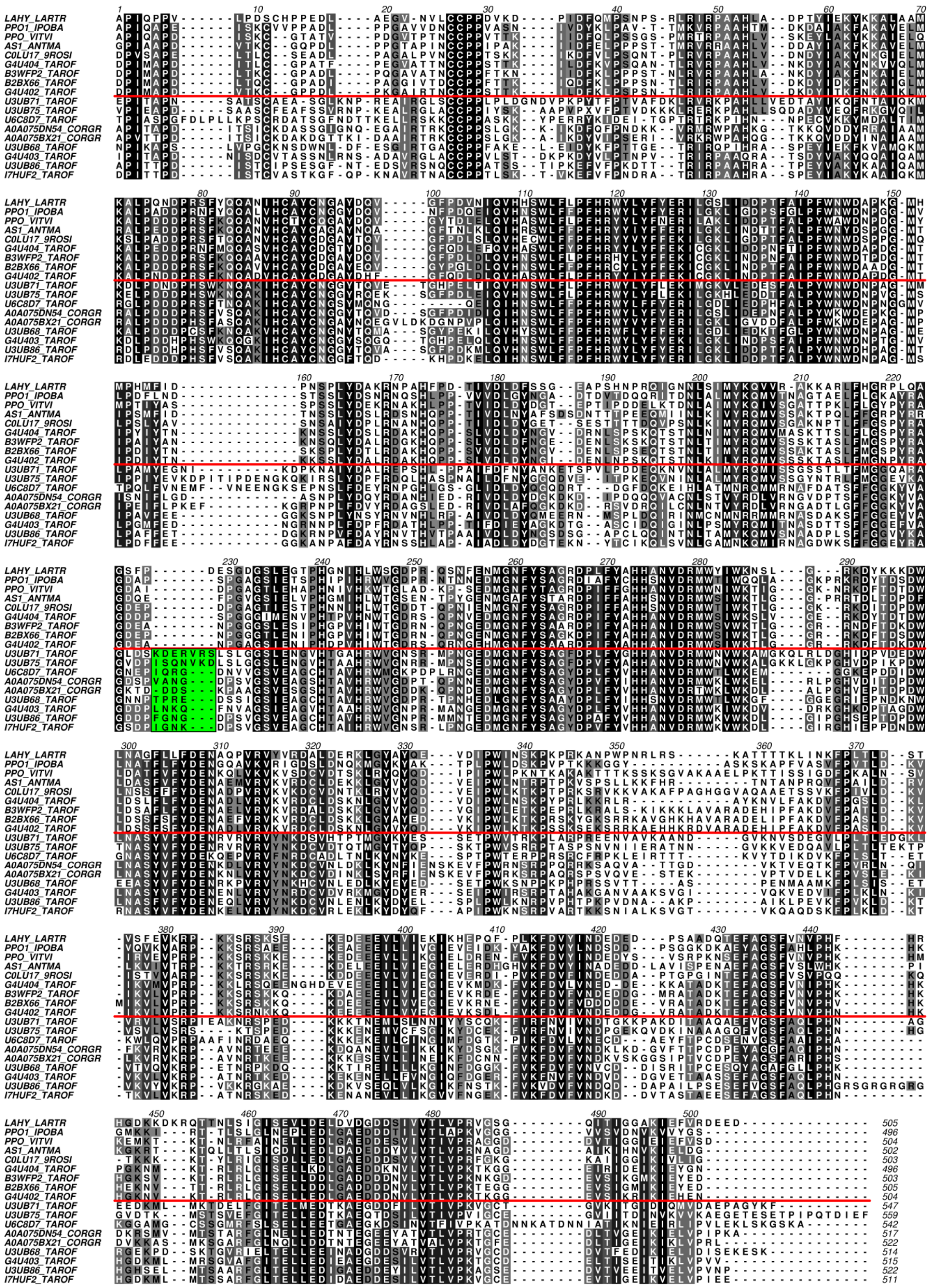

Supplement: Supplementary file 3 — Supplementary material 3 (PDF 1342 kb) [file 425_2015_2261_MOESM3_ESM.pdf]
