# Supplementary material for: Latent and active aurone synthase from petals of C. grandiflora: a polyphenol oxidase with unique characteristics
Source: Planta. 2015 Feb 20;242(3):519–37. doi: 10.1007/s00425-015-2261-0 (PMC4540782; doi:10.1007/s00425-015-2261-0)
Supplement: Supplementary file 4 — Supplementary material 4 (PDF 35 kb) [file 425_2015_2261_MOESM4_ESM.pdf]

ONLINE RESOURCE

Latent and active aurone synthase from petals of *C. grandiflora*: a polyphenol oxidase with unique characteristics

Christian Molitor, Stephan Gerhard Mauracher, Sanela Pargan, Rupert L. Mayer, Heidi Halbwirth, Annette Rompel*

* Corresponding author: Annette Rompel, Institut für Biophysikalische Chemie, Fakultät für Chemie, Universität Wien, Althanstraße 14, 1090 Wien, Austria.

E-mail: annette.rompel@univie.ac.at

Online Resource Table S1 Masses determined from deconvoluted mass spectra of active *cg*AUS samples under non-reducing and reducing conditions.Just prior to measurements acetonitrile and formic acid were added to a final concentration of 25 % (v/v) acetonitrile and 0.05 % (v/v) formic acid. Non-reducing conditions: samples were desalted to 10 mM ammonium acetate pH 5.0. Reducing conditions: samples were incubated for 45 min at 50 °C in 20 mM ammonium acetate pH 7.5, supplemented with 50 mM DTT. Due to overlapping peaks, shifts of the experimental masses to higher or to lower values are expected

Sample	Species	M (exp.) [Da]	Deduced sequence	M (calc.) [Da]
**2**	H	41140.3 ± 0.4	API - IENSKE // TTPCDPEYAGG	41140.90
	J	41386.3 ± 0.6	API - IENSKE // VFTTPCDPEYAGG	41387.21
	K	41443.9 ± 0.5	API - IENSKE // GVFTTPCDPEYAGG	41444.26
	N	41558.6 ± 0.2	API - IENSKE // DGVFTTPCDPEYAGG	41559.35
	P	41686.9 ± 0.3	API - IENSKE // KDGVFTTPCDPEYAGG	41687.53
	R	41705.1 ± 0.1	API - IENSKE // DGVFTTPCDPEYAGGF	41706.53
	I	41219.8 ± 0.7	API - IENSKE + X // TTPCDPEYAGG	41220.9
	L	41463.1 ± 1.0	API - IENSKE + X // VFTTPCDPEYAGG	41467.21
	M	41523.8 ± 0.1	API - IENSKE + X // GVFTTPCDPEYAGG	41524.26
	O	41638.6 ± 0.2	API - IENSKE + X // DGVFTTPCDPEYAGG	41639.35
	Q	41767.0 ± 0.3	API - IENSKE + X // KDGVFTTPCDPEYAGG	41767.53
	S	41784.7 ± 0.2	API - IENSKE + X // DGVFTTPCDPEYAGGF	41786.53
**2 reduced**	T	40035.93 ± 0.2	API - IENSKE	40036.79
	U	40115.95 ± 0.3	API - IENSKE + X	40116.79
	V	1110.4418 MH^{+1} (mono)	TTPCDPEYAGG	1110.4408 MH^{+1} (mono)
	W	678.7958 MH^{+2} (mono)	VFTTPCDPEYAGG	678.7925 MH^{+2} (mono)
	X	707.3051 MH^{+2} (mono)<br>1413.5996 MH^{+1} (mono)	GVFTTPCDPEYAGG	707.3032 MH^{+2} (mono)<br>1413.5991 MH^{+1} (mono)
	Y	764.8195 MH^{+2} (mono)<br>1528.6400 MH^{+1} (mono)	DGVFTTPCDPEYAGG	764.8167 MH^{+2} (mono)<br>1528.6261 MH^{+1} (mono)
	Z	828.8687 MH^{+2} (mono)	KDGVFTTPCDPEYAGG	828.6841 MH^{+2} (mono)
	AA	838.3464 MH^{+2} (mono)	DGVFTTPCDPEYAGGF	838.9085MH^{+2} (mono)
**3**	AB	41386.3 ± 1.0	API - IENSKE // VFTTPCDPEYAGG	41387.21
	AC	41444.0 ± 0.6	API - IENSKE // GVFTTPCDPEYAGG	41444.26
	AF	41558.9 ± 0.3	API - IENSKE // DGVFTTPCDPEYAGG	41559.35
	AH	41686.7 ± 0.3	API - IENSKE // KDGVFTTPCDPEYAGG	41687.53
	AI	41705.5 ± 0.9	API - IENSKE // DGVFTTPCDPEYAGGF	41706.53
	AL	41800.3 ± 0.3	API - IENSKE // LKDGVFTTPCDPEYAGG	41800.69
	AD	41466.3 ± 0.9	API - IENSKE + X // VFTTPCDPEYAGG	41467.21
	AE	41524.0 ± 0.7	API - IENSKE + X // GVFTTPCDPEYAGG	41524.26
	AG	41638.7 ± 0.2	API - IENSKE + X // DGVFTTPCDPEYAGG	41639.35
	AJ	41767.1 ± 0.4	API - IENSKE + X // KDGVFTTPCDPEYAGG	41767.53
	AK	41784.6 ± 0.7	API - IENSKE + X // DGVFTTPCDPEYAGGF	41786.53
	AM	41880.6 ± 0.7	API - IENSKE + X // LKDGVFTTPCDPEYAGG	41880.69

Sample	Species	M (exp.) [Da]	Deduced sequence	M (calc.) [Da]
**3 reduced**	AN	39778.2 ± 1.3	API-IENS	39779.50
	AO	39907.2 ± 0.7	API-IENSK	39907.67
	AP	40036.2 ± 0.2	API-IENSKE	40036.79
	AQ	40116.1 ± 0.5	API - IENSKE + X	40116.79
	AR	678.7922 MH^{+2} (mono)	VFTTPCDPEYAGG	678.7925 MH^{+2} (mono)
	AS	707.3058 MH^{+2} (mono)<br>1413.5996 MH^{+1} (mono)	GVFTTPCDPEYAGG	707.3032 MH^{+2} (mono)<br>1413.5991 MH^{+1} (mono)
	AT	764.821 MH^{+2} (mono)<br>1528.6301 MH^{+1} (mono)	DGVFTTPCDPEYAGG	764.8167 MH^{+2} (mono)<br>1528.6261 MH^{+1} (mono)
	AU	828.868 MH^{+2} (mono)	KDGVFTTPCDPEYAGG	828.6841 MH^{+2} (mono)
	AV	838.3513 MH^{+2} (mono)	DGVFTTPCDPEYAGGF	838.9085MH^{+2} (mono)
	AW	885.4104 MH^{+2} (mono)	LKDGVFTTPCDPEYAGG	885.4062 MH^{+2} (mono)
**4**	AX	41444.4 ± 0.9	API-IENSKE // GVFTTPCDPEYAGG	41444.26
	AZ	41558.8 ± 0.2	API - IENSKE // DGVFTTPCDPEYAGG	41559.35
	BB	41656.1 ± 0.6	API - IENSKEV // DGVFTTPCDPEYAGG	41658.49
	BC	41705.1 ± 0.7	API - IENSKE // DGVFTTPCDPEYAGGF	41706.53
	BF	41804.3 ± 0.5	API - IENSKEVF // DGVFTTPCDPEYAGG	41805.66
	AY	41523.1 ± 1.1	API-IENSKE + X // GVFTTPCDPEYAGG	41524.26
	BA	41638.9 ± 0.2	API - IENSKE + X // DGVFTTPCDPEYAGG	41639.35
	BD	41738.0 ± 0.2	API - IENSKEV + X // DGVFTTPCDPEYAGG	41738.49
	BE	41785.8 ± 0.7	API - IENSKE + X // DGVFTTPCDPEYAGGF	41786.53
	BG	41885.1 ± 0.2	API - IENSKEVF + X // DGVFTTPCDPEYAGG	41885.66
**4 reduced**	BH	40036.2 ± 0.1	API-IENSKE	40036.79
	BJ	40133.8 ± 0.6	API-IENSKEV	40135.92
	BL	40282.7 ± 0.6	API-IENSKEVF	40283.1
	BN	40378.1 ± 0.4	API-IENSKEVFP	40380.21
	BI	40116.3 ± 0.4	API-IENSKE + X	40116.79
	BK	40215.5 ± 0.3	API-IENSKEV + X	40215.92
	BM	40362.8 ± 0.4	API-IENSKEVF + X	40363.1
	BO	40459.7 ± 1.0	API-IENSKEVFP + X	40460.21
	BP	707.3080 MH^{+2} (mono))	GVFTTPCDPEYAGG	707.3032 MH^{+2} (mono)
	BQ	764.8204 MH^{+2} (mono)<br>1528.6302 MH^{+1} (mono)	DGVFTTPCDPEYAGG	764.8167 MH^{+2} (mono)<br>1528.6261 MH^{+1} (mono)
	BR	838.8579 MH^{+2} (mono)	DGVFTTPCDPEYAGGF	838.9085 MH^{+2} (mono)
