# Supplementary material for: Latent and active aurone synthase from petals of C. grandiflora: a polyphenol oxidase with unique characteristics
Source: Planta. 2015 Feb 20;242(3):519–37. doi: 10.1007/s00425-015-2261-0 (PMC4540782; doi:10.1007/s00425-015-2261-0)
Supplement: Supplementary file 5 — Supplementary material 5 (PDF 21 kb) [file 425_2015_2261_MOESM5_ESM.pdf]

ONLINE RESOURCE

Latent and active aurone synthase from petals of *C. grandiflora*: a polyphenol oxidase with unique characteristics

Christian Molitor, Stephan Gerhard Mauracher, Sanela Pargan, Rupert L. Mayer, Heidi Halbwirth, Annette Rompel*

* Corresponding author: Annette Rompel, Institut für Biophysikalische Chemie, Fakultät für Chemie, Universität Wien, Althanstraße 14, 1090 Wien, Austria.
E-mail: annette.rompel@univie.ac.at

Online Resource Table S2 List of peptides found by nanoLC-ESI-MS/MS protein identification experiments. **a** Peptides of *cg*AUS1 (A0A075DN54) found in active *cg*AUS (sample 1). Sequence coverage: 77 % (272/350 amino acids). **b** Peptides of *cg*AUS2 (A0A075BWS7) found in active *cg*AUS (sample 1). **c** Peptides of *cg*AUS1 (A0A075DN54) found in latent *cg*AUS (sample 5). Sequence coverage: 85 % (441/517 amino acids). **d** Peptides of *cg*AUS2 (A0A075BWS7) found in latent *cg*AUS (sample 5). Unique: peptides that discriminate between *cg*AUS1 and *cg*AUS2

a

Start-End	Sequence	Modifications	XCorr	Delta mass [ppm]	Peptide mass [M+H]+	Charge	Unique
**1-13**	APITAPDITSIcK	C12(Carbamidomethyl)	4.23	0.43	1386.73039	2	yes
**1-27**	APITAPDITSIcKDASSGIGNQEGAIR	C12(Carbamidomethyl)	6.87	2.05	2742.37845	3	yes
**1-29**	APITAPDITSIcKDASSGIGNQEGAIRTR	C12(Carbamidomethyl)	6.09	0	2999.52163	4	yes
**14-27**	DASSGIGNQEGAIR		4.89	0.14	1374.66106	2	yes
**14-29**	DASSGIGNQEGAIRTR		3.5	-0.18	1631.80937	2	yes
**30-38**	KccPPSLGK	C2(Carbamidomethyl); C3(Carbamidomethyl)	3.16	1.1	1046.51335	2	no
**39-49**	KIKDFQFPNDK		3.15	0.25	1379.73218	3	yes
**40-49**	IKDFQFPNDK		3.42	0.31	1251.63725	2	yes
**42-50**	DFQFPNDKK		2.57	0.63	1138.55351	2	yes
**42-52**	DFQFPNDKKVR		3	0.21	1393.72263	3	yes
**71-91**	AIAAMRALPDDDPRSFVSQAK		3.14	1.01	2259.15744	3	yes
**77-91**	ALPDDDPRSFVSQAK		3.48	-0.86	1645.8167	2	yes
**127-134**	WYLYFYER		2.64	-0.03	1239.5833	2	yes
**135-151**	ILGSLIDEPNFALPYWK		4.86	0.27	1976.05339	2	yes
**157-177**	GMPISNIFLGDASNPLYDQYR		5.27	0.89	2371.14092	2	yes
**157-185**	GMPISNIFLGDASNPLYDQYRDANHIEDR		5.61	-0.51	3321.55776	4	yes
**178-194**	DANHIEDRIVDLDYDGK		4.74	-0.22	1987.93521	3	yes
**178-196**	DANHIEDRIVDLDYDGKDK		4.02	0.3	2231.05825	4	yes
**186-196**	IVDLDYDGKDK		2.82	0.54	1280.63762	2	yes
**186-213**	IVDLDYDGKDKDIPDQQQVAcNLSTVYR	C21(Carbamidomethyl)	7.32	0.53	3268.58091	3	yes
**195-213**	DKDIPDQQQVAcNLSTVYR	C12(Carbamidomethyl)	5.6	0.25	2250.08257	2	yes
**197-213**	DIPDQQQVAcNLSTVYR	C10(Carbamidomethyl)	5.14	-0.71	2006.95867	2	yes
**218-229**	NGVDPTSFFGGK		3.65	-0.76	1225.58391	2	yes
**230-257**	yVAGDSPVANGDPSVGSVEAGSHTAVHR	Y1(Phospho)	6.26	-2.78	2816.25583	3	yes
**230-257**	yVAGDSPVANGDPSVGSVEAGSHTAVHR	Y1(Sulfo)	6.45	-3.56	2816.24411	3	yes
**230-257**	YVAGDSPVANGDPSVGSVEAGSHTAVHR		7.64	0.07	2736.29752	3	yes
**258-291**	WVGDPTQPNNEDMGNFYSAGYDPVFYIHHANVDR		6.05	-0.18	3925.72939	4	yes
**298-324**	ELRLPGHVDITDPDWLNASYVFYDENK		6.54	-1.65	3206.53774	4	yes
**298-328**	ELRLPGHVDITDPDWLNASYVFYDENKDLVR		8.09	1.6	3689.82949	4	yes
**301-324**	LPGHVDITDPDWLNASYVFYDENK		6.31	0.66	2808.31711	3	yes
**301-328**	LPGHVDITDPDWLNASYVFYDENKDLVR		7.99	-1.34	3291.5914	3	yes
**329-339**	VYNKDcVNLDK	C6(Carbamidomethyl)	3.28	1	1367.66379	3	yes
**329-341**	VYNKDcVNLDKLK	C6(Carbamidomethyl)	5.18	1.06	1608.84317	3	yes
**333-341**	DcVNLDKLK	C2(Carbamidomethyl)	3.23	-0.1	1104.57172	3	yes
**402-416**	TEEEKDQANEVLLIK		5.88	0.26	1758.91252	2	yes
**402-417**	TEEEKDQANEVLLIKK		6.39	-1.9	1887.00345	3	yes
**428-435**	FDVFVNDK		2.81	0.5	983.48381	2	no
**497-508**	TGcEDLTVGEIK	C3(Carbamidomethyl)	3.7	-0.23	1321.63017	2	yes

b

Start-End	Sequence	Modifications	XCorr	Delta mass [ppm]	Peptide mass [M+H]+	Charge	Unique
1-13	APVTTPDITSIcK	C12(Carbamidomethyl)	3.41	-3.34	1402.72002	2	yes
30-38	KccPPSLGK	C2(Carbamidomethyl); C3(Carbamidomethyl)	3.16	1.1	1046.51335	2	no
193-205	IVDLAFQGKDKDR		4.12	-0.24	1504.81154	4	yes
335-345	VYNKDcVDINK	C6(Carbamidomethyl)	3.01	1	1367.66379	3	yes
434-441	FDVFVNDK		2.81	0.5	983.48381	2	no

c

Start-End	Sequence	Modifications	XCorr	Delta mass [ppm]	Peptide mass [M+H]+	Charge	Unique
1-13	APITAPDITSIcK	C12(Carbamidomethyl)	4.18	-0.71	1386.72881	2	yes
1-27	APITAPDITSIcKDASSGIGNQEGAIR	C12(Carbamidomethyl)	7.73	2.59	2742.37992	3	yes
1-29	APITAPDITSIcKDASSGIGNQEGAIRTR	C12(Carbamidomethyl)	6.14	-0.73	2999.51943	4	yes
14-27	DASSGIGNQEGAIR		4.95	-1.91	1374.65825	2	yes
14-29	DASSGIGNQEGAIRTR		3.35	-0.3	1631.80918	3	yes
39-49	KIKDFQFPNDK		3.14	-0.28	1379.73145	3	yes
40-49	IKDFQFPNDK		3.5	0.5	1251.6375	2	yes
40-50	IKDFQFPNDKK		3.31	-0.09	1379.73172	3	yes
53-62	MRWPAHKGTK		3.19	-0.6	1211.64594	3	yes
64-70	KQVDDYRR		3.07	-0.71	1079.55851	3	yes
71-84	AIAAMRALPDDDPR		3.02	-0.72	1511.7625	2	yes
71-91	AIAAMRALPDDDPRSFVSQAK		4.11	-1.66	2259.1514	3	yes
77-91	ALPDDDPRSFVSQAK		3.37	-0.49	1645.81731	2	yes
127-134	WYLYFYER		2.69	-0.12	1239.58318	2	yes
135-151	ILGSLIDEPNFALPYWK		4.89	0.15	1976.05315	2	yes
157-177	GMPISNIFLGDASNPLYDQYR		5.33	2.33	2371.14433	2	yes
157-185	GMPISNIFLGDASNPLYDQYRDANHIEDR		5.67	0.69	3321.56174	3	yes
178-185	DANHIEDR		2.62	1.29	969.43974	2	yes
186-194	IVDLDYDGK		2.54	1.11	1037.51616	2	yes
186-196	IVDLDYDGKDK		3.03	0.46	1280.63752	3	yes
186-213	IVDLDYDGKDKDIPDQQQVAcNLSTVYR	C21(Carbamidomethyl)	7.92	0.08	3268.57944	3	yes
195-213	DKDIPDQQQVAcNLSTVYR	C12(Carbamidomethyl)	5.65	0.47	2250.08306	2	yes
197-213	DIPDQQQVAcNLSTVYR	C10(Carbamidomethyl)	5.15	2.7	2006.9655	2	yes
197-217	DIPDQQQVAcNLSTVYRDLVR	C10(Carbamidomethyl)	3.48	0.34	2490.24149	3	yes
218-229	NGVDPTSFFGGK		3.51	0.24	1225.58513	2	yes
218-257	NGVDPTSFFGGKYVAGDSPVANGDPSVGSVEAG SHTAVHR		6.41	0.39	3942.86587	4	yes
230-257	yVAGDSPVANGDPSVGSVEAGSHTAVHR	Y1(Phospho)	7.12	-3.76	2816.25309	3	yes
230-257	yVAGDSPVANGDPSVGSVEAGSHTAVHR	Y1(Sulfo)	7.12	-0.38	2816.25309	3	yes
230-257	YVAGDSPVANGDPSVGSVEAGSHTAVHR		7.84	-0.93	2736.29477	3	yes
258-291	WVGDPTQPNNEDMGNFYSAGYDPVFYIHHANV DR		6.65	1.69	3925.73673	3	yes
298-324	ELRLPGHVDITDPDWLNASYVFYDENK		6.9	-0.28	3206.54213	4	yes
298-328	ELRLPGHVDITDPDWLNASYVFYDENKDLVR		8.58	-0.19	3689.8229	4	yes
301-324	LPGHVDITDPDWLNASYVFYDENK		7.1	0.25	2808.31597	2	yes
301-328	LPGHVDITDPDWLNASYVFYDENKDLVR		8.9	1.11	3291.59946	3	yes
329-341	VYNKDcVNLDKLK	C6(Carbamidomethyl)	5.21	1.45	1608.8438	2	yes
333-341	DcVNLDKLK	C2(Carbamidomethyl)	3.38	-0.22	1104.57158	2	yes
333-349	DcVNLDKLKYNFIENSK	C2(Carbamidomethyl)	4.96	-2.22	2100.03843	3	yes
340-349	LKYNFIENSK		3.19	-1.89	1255.66578	3	yes
342-349	YNFIENSK		2.56	0.13	1014.48924	2	yes
342-355	YNFIENSKEVFPWR		4.61	-1.23	1828.89951	3	yes
364-375	KSAQVATTGDVK		4.05	-1.83	1204.65105	2	yes
365-375	SAQVATTGDVK		2.8	0.79	1076.55913	2	yes
365-381	SAQVATTGDVKTVEQTK		5.12	-0.01	1762.91819	3	yes
365-385	SAQVATTGDVKTVEQTKFPVR		6.87	-1.15	2262.20633	3	yes

376-385	TVEQTKFPVR		3.21	1.32	1204.67009	2	yes
376-391	TVEQTKFPVRLNQIFK		5.19	0.16	1948.10185	2	yes
386-393	LNQIFKVR		2.8	-1.63	1017.61876	2	yes
396-416	RPAVNRTEEEKDQANEVLLIK		6.57	-0.19	2452.31508	4	yes
402-416	TEEEKDQANEVLLIK		6.56	1.16	1758.91411	2	yes
402-417	TEEEKDQANEVLLIKK		6.55	-0.54	1887.00602	3	yes
407-416	DQANEVLLIK		3.06	-0.72	1142.64079	2	yes
407-417	DQANEVLLIKK		3.77	1.18	1270.73808	2	yes
418-427	IKYDSGKFVK		3.34	0.87	1184.66846	3	yes
420-435	YDSGKFVKFDVFVNDK		4.7	-0.31	1907.95328	3	yes
425-435	FVKFDVFVNDK		4.02	-2.42	1357.71184	2	yes
425-437	FVKFDVFVNDKLK		4.22	-1.97	1598.89103	3	yes
428-435	FDVFVNDK		3.14	-1.42	983.48192	2	no
428-437	FDVFVNDKLK		2.67	0.13	1224.66252	2	yes
436-462	LKDGVFTTPcDPEYAGGFAQIPHNDKR	C10(Carbamidomethyl)	6.2	-0.7	3033.45034	4	yes
438-461	DGVFTTPcDPEYAGGFAQIPHNDK	C8(Carbamidomethyl)	4.95	-0.27	2636.1716	3	yes
438-462	DGVFTTPcDPEYAGGFAQIPHNDKR	C8(Carbamidomethyl)	6.24	-1.71	2792.26865	3	yes
472-496	FGLNELLEDTNTEGEEYATVTLVPR		7.2	0.84	2810.37554	2	yes
497-508	TGcEDLTVGEIK	C3(Carbamidomethyl)	4.1	1.16	1321.63201	2	yes
497-516	TGcEDLTVGEIKIELVPIPK	C3(Carbamidomethyl)	6.25	-2.24	2211.18926	2	yes
497-517	TGcEDLTVGEIKIELVPIPKA	C3(Carbamidomethyl)	6.55	0.6	2282.23271	2	yes
509-516	IELVPIPK		2.64	0.14	908.58171	2	yes
509-517	IELVPIPKA		2.65	0.62	979.61931	2	yes

d

Start-End	Sequence	Modifications	XCorr	Delta mass [ppm]	Peptide mass [M+H]+	Charge	Unique
1-13	APVTTPDITSIcK	C12(Carbamidomethyl)	2.91	-0.21	1402.72441	2	yes
30-38	KccPPSLGK	C2(Carbamidomethyl); C3(Carbamidomethyl)	3.3	1.45	1046.51372	2	no
434-441	FDVFVNDK		3.14	-1.42	983.48192	2	no
